# CsgA gatekeeper residues control nucleation but not stability of functional amyloid

**DOI:** 10.1002/pro.5178

**Published:** 2024-09-20

**Authors:** William P. Olsen, Gaston Courtade, Samuel Peña‐Díaz, Madhu Nagaraj, Thorbjørn V. Sønderby, Frans A. A. Mulder, Mette G. Malle, Daniel E. Otzen

**Affiliations:** ^1^ Interdisciplinary Nanoscience Center (iNANO) Aarhus University Aarhus C Denmark; ^2^ Sino‐Danish College (SDC) University of Chinese Academy of Sciences Beijing China; ^3^ Norwegian Biopolymer Laboratory (NOBIPOL), Department of Biotechnology and Food Science NTNU Norwegian University of Science and Technology Trondheim Norway; ^4^ Institute of Biochemistry Johannes Kepler University Linz Austria; ^5^ Department of Chemistry Aarhus University Aarhus C Denmark

**Keywords:** CHAPS, CsgA, curli, functional amyloid, gatekeepers, NMR, ThT

## Abstract

Functional amyloids, beneficial to the organism producing them, are found throughout life, from bacteria to humans. While disease‐related amyloids form by uncontrolled aggregation, the fibrillation of functional amyloid is regulated by complex cellular machinery and optimized sequences, including so‐called gatekeeper residues such as Asp. However, the molecular basis for this regulation remains unclear. Here we investigate how the introduction of additional gatekeeper residues affects fibril formation and stability in the functional amyloid CsgA from *E. coli*. Step‐wise introduction of additional Asp gatekeepers gradually eliminated fibrillation unless preformed fibrils were added, illustrating that gatekeepers mainly affect nucleus formation. Once formed, the mutant CsgA fibrils were just as stable as wild‐type CsgA. HSQC NMR spectra confirmed that CsgA is intrinsically disordered, and that the introduction of gatekeeper residues does not alter this ensemble. NMR‐based Dark‐state Exchange Saturation Transfer (DEST) experiments on the different CsgA variants, however, show a decrease in transient interactions between monomeric states and the fibrils, highlighting a critical role for these interactions in the fibrillation process. We conclude that gatekeeper residues affect fibrillation kinetics without compromising structural integrity, making them useful and selective modulators of fibril properties.

## 1 INTRODUCTION

The widespread presence of amyloid fibrils in Nature reveals that the ability to fibrillate is an intrinsic property of amino acid‐based peptide chains (Ke et al., 2020; Knowles et al., 2014; Knowles & Mezzenga, 2016). The amyloid fold is known for its high stability, physical and chemical resistance, and self‐templating replication (Knowles & Mezzenga, 2016). Amyloid fibrils are known for their role in mammalian neurodegenerative diseases such as Alzheimer's, Parkinson's, and Mad Cow disease (Wong & Krainc, 2017). In pathology, a common feature for these proteins is a runaway aggregation mechanism which becomes increasingly harder for the cellular quality control machinery to deal with as the fibrils grow and multiply (Ke et al., 2020; Meisl et al., 2022; Wong & Krainc, 2017). In contrast, functional amyloids have evolved to utilize the unique properties of amyloids for the benefit of the host (Badtke et al., 2009; Evans et al., 2018; Fowler et al., 2007; Otzen & Riek, 2019). To achieve this, functional amyloids are formed in a very tightly controlled process that ensures that aggregation only occurs at the right time and place. Functional amyloids, in addition to having the support of chaperones and translocation pores, also display far less replication through secondary (and less easily controlled) processes such as secondary nucleation and fragmentation (Meisl et al., 2022), which are much more prevalent in pathological amyloids (Cohen et al., 2013; Meisl et al., 2014). Thus, functional amyloids achieve replication rates appropriate to the growth rates of their biological hosts (Meisl et al., 2022). It is likely that evolutionary pressure has guided the functional amyloids to suppress uncontrolled self‐replication, but the molecular mechanism behind it is still unclear.

Curli is one of the most studied functional amyloids and an important component of *E. coli* biofilm formation, providing physical and chemical protection to bacterial communities (Barnhart & Chapman, 2006; Chapman, 2002). The curli system consists of at least six different proteins, with CsgA as the main constituent of curli fibrils while the other five help facilitate safe, efficient, and correct fibril formation by CsgA (Barnhart & Chapman, 2006; Chapman, 2002). The minor component of curli fibrils, CsgB, acts as tethering and nucleation sites for CsgA, anchoring the fibrils to the outer cell membrane (Hammer et al., 2007). CsgA remains unstructured in vivo in the absence of CsgB (Hammar et al., 1996). CsgD is a transcriptional activator for the csgBA operon (Barnhart & Chapman, 2006). CsgG, CsgE, CsgF, and CsgC are all mediator proteins, acting as transport proteins and chaperones (Chapman, 2002). CsgG is thought to be responsible for the transport of CsgA, CsgB, and CsgF across the outer membrane (Nenninger et al., 2009). CsgF interacts with CsgG at the outer membrane and acts as a tethering point for CsgB (Robinson et al., 2006). CsgE interacts with CsgG in the periplasmic space and together with CsgC is responsible for stabilizing CsgA, CsgB, and CsgF through a chaperone‐like mechanism until properly translocated (Nenninger et al., 2009).

**FIGURE 1:**
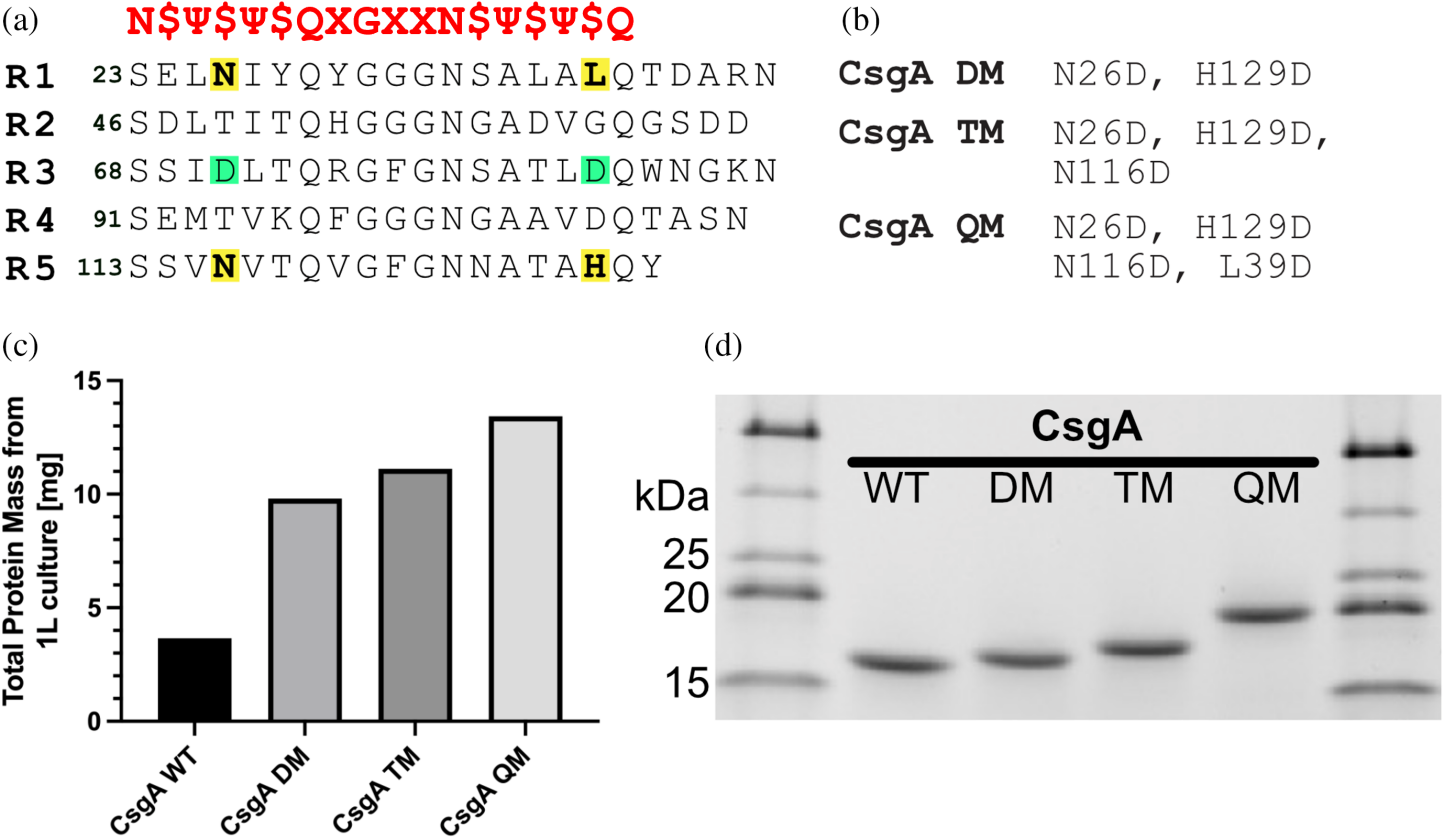
Asp gatekeeper residues originally only present in R3 were introduced into R1 and R5 through site‐directed mutagenesis. (a) Alignment of the five repeats of CsgA responsible for forming the amyloid core. Asp gatekeeper residues of R3 are highlighted in green and residues targeted for site‐directed mutagenesis in R1 and R5 are highlighted in yellow. The red sequence above the five repeats is the consensus sequence obtained by comparison of >40,000 different CsgA sequences (Sleutel et al., 2023), where Ψ are hydrophobic residues facing into the core of the amyloid structure alternating with surface exposed ($) residues. (b) Residue changes found in CsgA DM, TM, and QM. (c) The amount of purified recombinant CsgA produced per L culture increases significantly with mutation. (d) SDS‐PAGE gel highlights the difference in migration behavior of wt CsgA, DM, TM, and QM.

Besides help from ancillary proteins, CsgA is also optimized for fibrillation through its sequence. The amyloid core of CsgA (i.e., the whole of the mature protein except for a 22‐residue N‐terminal stretch which is not structured in the final fibril (Bu et al., 2024; Sleutel et al., 2023; Tian et al., 2015)), consists of five imperfect repeats, all 19–23 amino acids in length and sharing at least 30% amino acid identity with the consensus sequence Ser‐X_5_‐Gln‐X_4_‐Asn‐X_5_‐Gln (Wang et al., 2010). Each repeat forms a β‐hairpin in which the fibrillar structures stack on top of each other to form a very compact amyloid repeat unit (Tian et al., 2015). A recent alignment of >40,000 different CsgA sequences identified an even more general amyloid motif across the bacterial metagenome where each strand in the repeat contains the motif N‐$‐Ψ‐$‐Ψ‐$‐Q, where Ψ are hydrophobic residues facing into the core of the amyloid structure alternating with surface exposed ($) residues (see alignment of this motif to CsgA from *E. coli* in Figure 1a). Repeats 1 (R1) and 5 (R5) are responsible for intermolecular interactions between both CsgA and CsgB, with repeats 2–4 sandwiched in between. While R2‐R4 are similar in sequence to R1 and R5, certain Asp and Gly residues found in R2‐R4 act as gatekeeper residues, making these repeats significantly less amyloidogenic and effectively restraining protein fibrillation. This avoids untimely and mislocated fibril formation in the cell (Wang et al., 2010). When introduced into position 116 and 129 in R5 (note we use the numbering for mature CsgA, excluding the first 20 residues comprising the signal peptide), Asp had a detrimental effect on the amyloidogenicity of CsgA while residues with similar properties as the wildtype residues showed minimal effect (Wang et al., 2010). Other studies of gatekeeper residues in aggregation‐prone regions (APR) have likewise concluded that electrostatic repulsion of charged residues is one of the main contributors to the suppression of aggregation (Beerten, Schymkowitz, & Rousseau, 2012; Houben et al., 2020; Kallberg et al., 2001; Monsellier & Chiti, 2007; Sant'Anna et al., 2014).

Given the role of R1 and R5 in intermolecular interactions, gatekeeper residues in these positions are expected to have a major impact on CsgA self‐assembly. Accordingly, here we investigate how the introduction of Asp gatekeeper residues into specific positions of the amyloidogenetic R1 and R5 of CsgA affects both the kinetics of amyloid formation and the structure and behavior of the resulting amyloid fibrils. As points of mutagenesis, we chose Asn26 and Leu39 in R1 and Asn116 and His129 in R5, all of which are predicted to be surface exposed according to the CsgA consensus motif (Figure 1a, b) and thus involved in inter‐monomer quaternary contacts. Additionally, we were interested in determining if and how multiple gatekeepers show cumulative effects which would shed more light on how the amyloidogenicity of functional amyloids in contrast to their pathological counterparts are fine‐tuned by their specific number of gatekeepers. We show that stepwise substitution of specific residues in R1 and R5 of CsgA with Asp gatekeeper residues gradually eliminates CsgA amyloidogenicity, primarily by inhibiting transient interactions and the primary nucleation step of the fibrillation mechanism. At the same time, NMR spectroscopy does not detect any appreciable change in structural propensity for the variants. These mutants only fibrillate in the presence of nucleation seeds or surface catalysis. Once formed, they are virtually indistinguishable from wt CsgA fibrils. Lastly, we utilize the monomeric stability of the CsgA QM variant in Dark‐state Exchange Saturation Transfer experiments to probe the interaction and on–off rates between CsgA and fibrils. This allows us to conclude that increasing the number of gatekeeping residues significantly reduces the fraction of the protein in the transiently bound state.

## 2 MATERIALS AND METHODS

### 2.1 Site‐directed mutagenesis

The residues were introduced via site‐directed mutagenesis using QuikChange (Braman et al., 1996). All CsgA constructs contain a C‐terminal His‐tag which facilitates purification by Ni‐NTA chromatography and which is retained in the subsequent studies.

### 2.2 Protein expression

Electro‐competent BL21‐DE3 *slyD* knockout cells were electroporated using 1 mm cuvettes in an Eporator (Eppendorf) at 1800 V for ≤5 ms and transformed with pET11a containing the CsgA genes. The wt CsgA gene was derived from the major curlin subunit from K12 (Uniprot ID: P28307), with the first 20 amino acids (signal peptide) removed. Sequences can be found in Data S1. Transformed cultures were plated on LB‐agar plates containing 100 μg/ml ampicillin and incubated overnight at 37°C. A single colony was used to inoculate a 10 ml preculture of 2xYT medium containing 100 μg/ml, which was incubated overnight at 37°C and then added to 1 L of 2xYT containing 100 μg/ml ampicillin and 0.5% glucose. The culture was incubated at 37°C with 150 RPM shaking, induced with 0.1 mM Isopropyl β‐d‐1‐thiogalactopyranoside (IPTG) at 1.7 OD_600_, and left to incubate for 2 h. The culture was harvested by centrifugation at 4000*g*, 4°C for 10 min. The resulting cell pellet was either frozen at −80°C for later purification or used straight away.

### 2.3 Protein purification

The cell pellet was dissolved by incubation overnight on a magnet stirrer at 4°C, in buffer A (7.5 M guanidinium hydrochloride (GdmCl) with 50 mM Tris, pH 7.4) using 100 ml buffer per 1 L of expression culture. The solution was centrifuged at 12.000 g, room temperature for 20 min to pellet insoluble cell debris, which was discarded. The supernatant was incubated with Ni‐NTA beads (2 ml per liter of expression culture) in a blue‐cap flask on a magnet stirrer for 1 h at room temperature. The Ni‐NTA beads were captured in a 50 ml Corning centrifuge tube by multiple rounds of gentle centrifugation at 1000 g for 1 min with slow deceleration. The supernatant was removed and discarded by careful decantation. The Ni‐NTA beads were then washed once with 50 ml buffer A and twice with buffer B (buffer A with 20 mM imidazole) by gently inverting the tube for 2 min followed by centrifugation and discarding the supernatant. The washed beads were transferred to a plastic gravity column with a ceramic filter bottom and 30 ml buffer B was added as a final wash. To elute bound CsgA, 20 ml buffer C (buffer A with 500 mM imidazole) was gently added to the top of the column and the flowthrough was collected in 1 ml fractions. CsgA concentration was measured on a DeNovix DS‐11 Nanodrop blanked with buffer C to eliminate imidazole absorption (0.5 M imidazole has an absorption of ca. 0.7 at 280 nm). All fractions containing CsgA were pooled, passed through a 0.2 μm filter, aliquoted, snap‐frozen in liquid nitrogen, and stored at −80°C. When needed for assays, CsgA was thawed under gentle shaking to fully dissolve all GdmCl crystals followed by desalting into 50 mM Tris, pH 7.4 using Cytiva PD MiniTrap desalting columns with Sephadex G‐25 resin. CsgA concentration was likewise measured on a DeNovix DS‐11 Nanodrop before use using ε_280_ = 11,460 M^−1^ cm^−1^.

### 2.4 SDS‐PAGE

Protein solutions were mixed with 2× BioRad Laemmli SDS‐PAGE Loading Buffer and loaded on BioRad TGX Stain‐Free 4–20% gels along with a BioRad Precision Plus Protein unstained ladder. The gels were run in Laemmli buffer at 125 V for 45 min. Protein bands were visualized with a GelDoc Go Gel Imaging System using the Stain‐free program.

### 2.5 ThT assay

Protein solutions were prepared in Eppendorf tubes and ThT was added to a final concentration of 20 μM before transfer to 96‐well plates. 100 μl solution was added to each well in half‐area plates while 200 μl was added to regular plates. All plates were sealed with MicroAmp Optical Adhesive Film prior to measuring. Fluorescence was measured every 10 min after 5 s of double orbital, 200 RPM shaking in a BMG Labtech CLARIOstar Plus microplate reader set to excitation at 448 nm and emission at 485 nm with a dichroic filter at 466.6 nm and gain set to 1500. All experiments were conducted at 37°C. Corning 96‐well Half Area Black, Clear Flat Bottom Polystyrene Nonbinding surface (NBS) plates were used for experiments that required non‐binding surface while Corning 96‐well Black, Clear Bottom Polystyrene Microplates were used for surface‐catalyzed experiments but were not washed prior to samples being added.

### 2.6 Fibril and fibril seeds preparation

Fibrils for TEM imaging, formic acid assays, The and seeding experiments were prepared by incubating high concentrations (>25 μM) of protein at 37°C in Eppendorf tubes for 48 h. For TEM imaging, fibrils were used without further treatment to avoid fragmentation. Fibrils needed for formic acid assays and seeding experiments were washed 3 times by 13,000 *g* centrifugation for 10 min followed by careful removal of supernatant and vigorous resuspension of the fibril pellet in fresh 50 mM Tris, pH 7.4 buffer. The protein concentration of the supernatant was measured to ensure that unpolymerized monomers were accounted for. Washed fibrils were sonicated immediately prior to use using a Fisherbrand 505 Sonicator with Probe in 5 cycles of 5 s bursts followed by 5 s of rest.

### 2.7 Formic acid assay

A sample of washed fibrils was sonicated according to the protocol described above and was immediately transferred to Eppendorf tubes. 50 μg of fibrils, calculated based on monomeric concentration, were used for each sample. All fibril samples were then centrifuged at 13,000 g for 10 min and the supernatant was very carefully removed and discarded. 50 μl of different concentrations of formic acid were added to the fibril pellet and carefully pipetted up and down followed by 10 s of gentle vortexing. The samples were incubated for 20 min at room temperature followed by centrifugation at 13,000 g for 10 min. 20 μl of supernatant were carefully pipetted from the top of the liquid and transferred into fresh Eppendorf tubes. Holes were poked in the lid and the samples were flash‐frozen in liquid nitrogen before being lyophilized for 90 min. The protein powder was carefully dissolved in 30 μl 2× BioRad Laemmli SDS‐PAGE Loading Buffer and loaded directly on a BioRad TGX Stain‐Free 4–20% SDS‐PAGE gel and run according to the protocol described above. SDS‐PAGE band intensity was analyzed using BioRad's Image Lab Software and all intensities were normalized to the 98% formic acid sample band. Data were fitted to the two‐state unfolding model described below (Christensen et al., 2020) using Prism 9 plotting software:

$$y_{\text{diss}}=\frac{100}{1+10^{\frac{-m_{\mathrm{FA}}*\left({\left[\mathrm{FA}\right]}^{50\%}-\left[\mathrm{FA}\right]\right)}{1.36}}}$$

The midpoint of denaturation [FA]^50%^, the free energy of unfolding Δ*G*, and the dependence of Δ*G* on [FA] (*m*_FA_) were obtained through this fitting.

### 2.8 TEM imaging

All TEM data were recorded on a Tecnai FEI F20 microscope using copper mesh 400 grids coated with carbon film from EMResolutions. All grids were prepared by placing 3 μl of sample on the grid for 2 min to allow for proper absorption onto the grid surface. Sample liquid was blotted away with filter paper followed immediately by staining with 3 μl 2% uranyl formate for 15 s. The uranyl formate was blotted away and the staining process was repeated two additional times after which the excess liquid was blotted and the grid air dried for 2 min before being put in storage.

### 2.9 TIRF imaging

To visualize the growth of fibrils of wt CsgA and CsgA DM, monomeric proteins were fluorescently labeled with Alexa Fluor 488 NHS Ester as follows: CsgA was desalted into Solution C (20 parts PBS, pH 7.4, and 1 part 0.2 M NaHCO_3_, pH 9.0) using a Cytiva PD MiniTrap desalting column with Sephadex G‐25 resin. The concentration of the desalted CsgA was immediately adjusted to 30 μM to reduce aggregation. 9.2 μl 7.8 mM Alexa Fluor 488 NHS Ester was added and mixed thoroughly before being wrapped in tin foil to protect the fluorescent dye from light, and then incubated at room temperature for 30 min. The solution was then desalted into PBS, pH 7.4, using a Cytiva PD10 desalting column with Sephadex G‐25 resin, taking care not to collect any free dye. The concentration of the conjugated CsgA protein was determined with a DeNovix DS‐11 Nanodrop using a CsgA extinction coefficient ε_280_ = 11,460 M^−1^ cm^−1^ and an Alexa 488 extinction coefficient ε_495_ = 73,000 M^−1^ cm^−1^. The CsgA solution was diluted to 1 μM in PBS and transferred into an Ibidi Sticky‐Slide VI 0.4 6‐channel slide glued onto a pre‐treated 170 μm thick microscope coverslip. Prior to this step, the coverslip had been cleaned by sonicating the glass slide 5 times 10 min in 2% Hellmanex solution and rinsed with ultrapure (MilliQ) water in between followed by 5 times sonication for 10 min in ultrapure water rinsed with ultrapure water in between sonication steps. Finally, the glass slides were sonicated one time in 96% ethanol and the ethanol was exchanged to new 96% ethanol and stored. The glass slide was dried with nitrogen and processed with plasma cleaning treatment for 5 min, after which it was incubated with a 1 g/L PLL‐g‐PEG solution for 30 min and rinsed with ultrapure water before being nitrogen dried and then attached to an Ibidi Sticky‐Slide VI 0.4 6 channel slide. All TIRF data were recorded on an ONI Nanoimager TIRF microscope. Laser intensity was adjusted to maximize contrast while autofocus and Z‐axis lock were activated during all experiments. Growth movies were recorded by taking images every 30 seconds for at least 3 h for wt CsgA and 8 h for CsgA DM for four fields of views per technical replicate enabled by the autofocus and Z‐axis lock. Growth data analysis was done in ImageJ by measuring the final length and time of growth for selected fibrils. Labeling with AlexFluor488 did not appreciably affect aggregation kinetics of CsgA (data not shown), probably because the two Lys residues in positions 89 and 96 are predicted to be in a turn and a surface‐exposed position, respectively.

### 2.10 NMR spectroscopy

To gain insights into the structural properties of CsgA, NMR experiments were conducted on CsgA QM in solution, using 200 μM ^15^N‐labeled CsgA in 20 mM Na_2_PO_4_, 10% D_2_O, pH 7.2. Spectra were assigned using HSQC, NOESY‐HSQC, and TOCSY‐HSQC spectra recorded at 283.2 K on a Bruker Avance III HD 950 MHz spectrometer using a 5 mm TCI liquid state cryoprobe. Spectra were processed in TOPSPIN and were analyzed and assigned using CCPN (Skinner et al., 2016). To gain insights into the dynamics of the interaction of CsgA with fibrils using relaxation and saturation transfer spectra, 30 μM solutions each of wt CsgA and the three mutants (DM, TM, QM) were prepared in 50 mM Na_2_PO_4_ and 10% D_2_O at pH 7.4. Following initial relaxation and saturation transfer measurements, samples containing wt CsgA and DM were allowed to fibrillate by incubation for 4 days at 6°C, in which measurements were taken at various time points. A final measurement was taken at the end of the experiment. CsgA TM and QM were unable to form fibrils in the same time span, and thus 5% preformed fibril seeds were added in order to obtain a similar dataset. Details of the NMR measurements are described below. These spectra were recorded at 298 K on a Bruker Ascend 800 MHz spectrometer with an Avance III HD console equipped with a 5 mm z‐gradient CP‐TCI (H/C/N) cryogenic probe at the NV‐NMR‐Centre/Norwegian NMR Platform, Norwegian University of Science and Technology (NTNU), Trondheim, Norway. Here two sets of measurements were made. (a) The apparent association rate, $k_{\mathrm{on}}^{\mathrm{app}}$, between CsgA proteins and fibrils was measured using changes in the R_2_ relaxation rate. CPMG spectra with water suppression by excitation sculpting were used to acquire ^1^H R_2_ rates in the presence and absence of fibrils. In this experiment, peak integrals (I) were obtained at eight values of τ from 0 to 250 ms, and fitted to $I=I_{0}e^{-R_{2}\tau }$ to extract R_2_. The maximum difference in R_2_ between the two conditions (with and without fibrils) was used to estimate $k_{\mathrm{on}}^{\mathrm{app}}$. The ^1^H R_1_ relaxation rate of CsgA QM was estimated using an inversion recovery experiment. (b) To investigate the “dark” state of CsgA transiently bound to the fibrils, ^1^H saturation transfer spectra were recorded as described (Fawzi et al., 2010). Briefly, 700 ms continuous wave irradiation at off‐resonance frequencies (15 offsets ranging from +35 to −35 kHz from the water resonance) with radiofrequency field strengths of 180 or 350 Hz were used to partially saturate the broad resonances of the fibril‐bound state. Saturation transfer from this “dark” state to the visible monomer was monitored by the decrease in intensity of an amide region (7.85–8.6 ppm) in the ^1^H NMR spectrum. To quantify the exchange dynamics between the “dark” and visible states, we analyzed the intensity profiles as a function of off‐resonance frequency. This analysis employed a two‐site exchange model as described (Fawzi et al., 2012), implemented in a Matlab script kindly provided by G. Marius Clore.

### 2.11 AlphaFold model generation

The AlphaFold model for CsgA QM was generated by inserting the amino acid sequence into the online Google Colab webpage (Jumper et al., 2021) and using the highest scoring mode:

https://colab.research.google.com/github/deepmind/alphafold/blob/main/notebooks/AlphaFold.ipynb.

The color‐coded PyMoL model of CsgA QM was generated using the following script:

*inFile = open*(*“Bfactor_N15_Urea.txt”, ‘r’*).

*stored.newB =* [].

*for line in inFile.readlines*(): *stored.newB.append*(*float*(*line*)).

*inFile.close*().

*alter CsgA, b = 0.0*.

*alter CsgA and n. CA, b = stored.newB.pop*(*0*).

*ramp_new colorbar, none*, [*−1.164, 0, 1.164*], [*blue, white, red*].

*spectrum b, blue_white_red, minimum = −1.164, maximum = 1.164*.

## 3 RESULTS

### 3.1 Introduction of gatekeeper residues in R1 and R5 completely inhibits fibril formation

Inspired by the pioneering work of Chapman and coworkers (Wang et al., 2010), we hypothesized that the introduction of Asp residues into two key locations each in R1 and R5 identified by alignment of the five repeats (shown in yellow in Figure 1a) would reduce CsgA's amyloidogenicity. Four Asp residues were introduced in a step‐wise manner to elucidate if and how the effect of multiple gatekeepers was cumulative. We constructed three mutants with increasing amounts of gatekeeper residues: CsgA Double Mutant (DM) with mutations N26D and H129D, CsgA Triple Mutant (TM) (DM plus N116D), CsgA Quadruple Mutant (QM) (TM plus L39D) (Figure 1b).

Each protein was purified according to the same expression and purification protocol. Interestingly, the protein yield generally increased with gatekeeper count (Figure 1c). On SDS‐PAGE, the four CsgA variants showed decreasing migration with an increasing number of gatekeepers (Figure 1d). We attribute this to a reduction in SDS‐binding (and thus loss of overall negative charge, which leads to a reduction in mobility in an electric field) upon introduction of SDS‐repelling anionic Asp residues.

**FIGURE 2:**
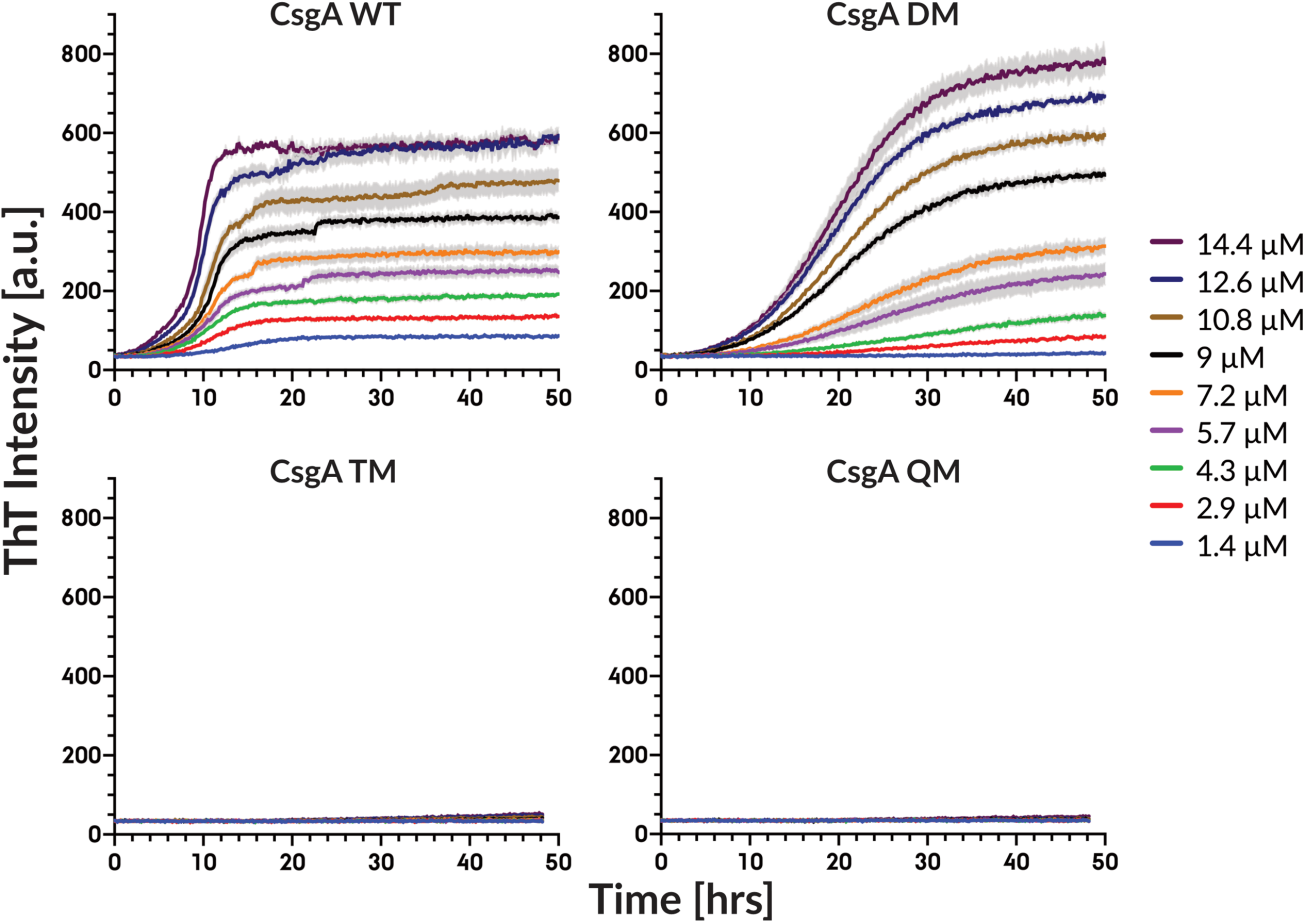
CsgA fibrillation propensity decreases as more Asp gatekeeper residues are introduced. All THT assays experiments were performed in low‐binding 96‐well plates. ThT fluorescence trace of fibrillation of wt CsgA, CsgA DM, CsgA TM, and CsgA QM. Error bars indicate 95% standard error of mean (SEM) with four technical repliates of each sample.

We used Thioflavin T (ThT) assays in low‐binding 96‐well plates to investigate the effect of gatekeeper residues in the 3 CsgA variants between 1.4 and 14.4 μM (Figure 2). The introduction of two gatekeeper residues (CsgA DM) significantly reduced the fibrillation propensity, giving both a large increase in the lag phase and a large decrease in the growth rate compared with wt CsgA fibrillation (Figure 2). The gatekeeper residues introduced in CsgA DM thus have a clear negative effect on both the primary nucleation as well as the elongation rates. Further addition of 1–2 gatekeeper residues in CsgA TM and QM completely inhibits fibrillation (Figure 2). The complete absence of fibrillation by both TM and QM prevented any conclusions at this stage about the additional effect of the fourth gatekeeper residue.

**FIGURE 3:**
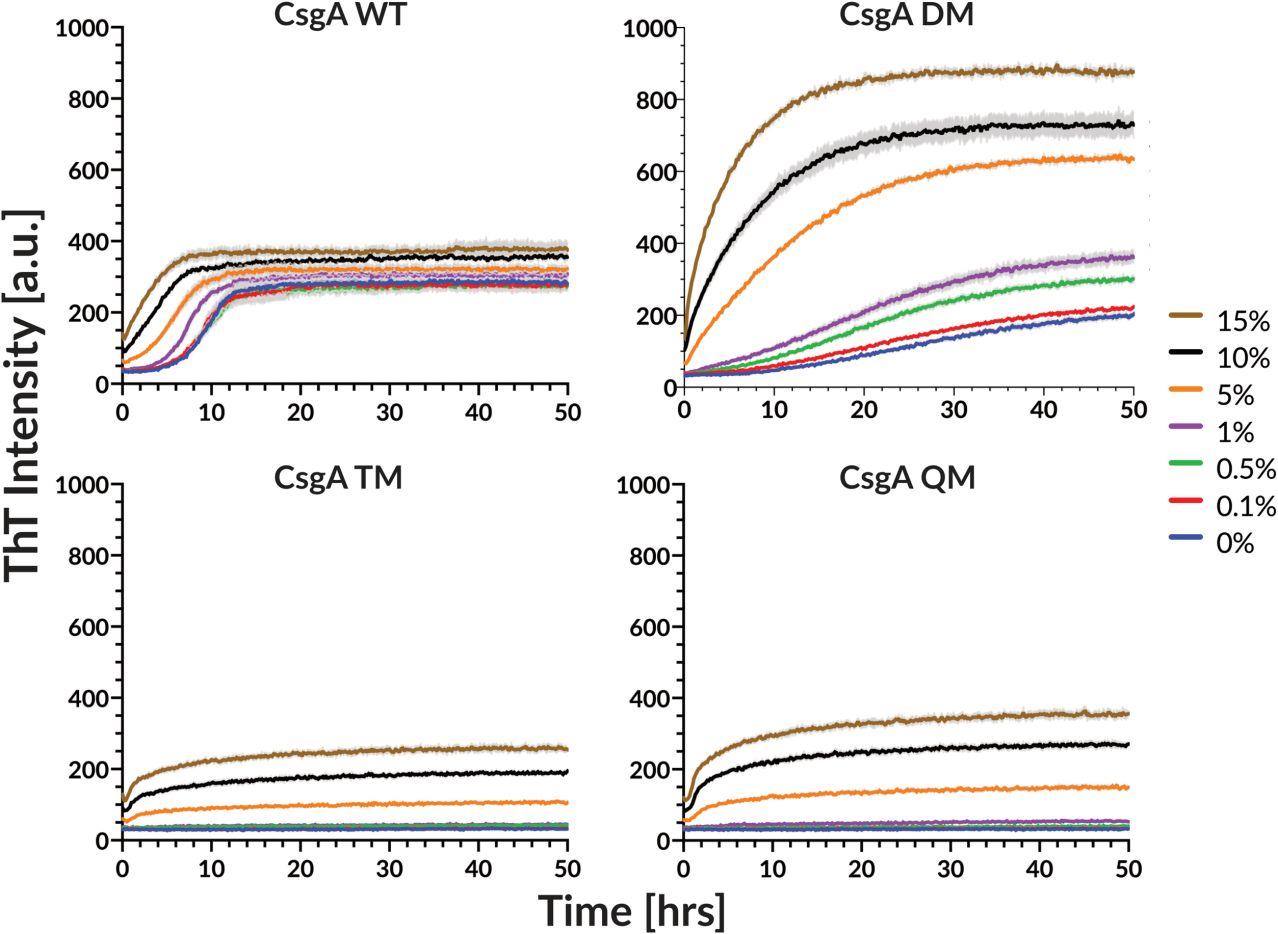
The addition of preformed fibril seeds accelerates the polymerization of all CsgA variants. Relatively high amounts of fibrils are required to observe significant effects. All polymerization experiments were performed at 7.2 μM total monomeric equivalent concentration with indicated amounts of preformed fibril seeds. Experiments were performed in low‐binding 96‐well plates. ThT fluorescence trace of self‐seeded polymerization of wt CsgA, CsgA DM, CsgA TM, and CsgA QM. Error bars indicate 95% standard error of mean (SEM) with four technical repliates of each sample.

Interestingly, all four variants readily formed fibrils in non‐coated 96‐well plates (Figure S1). We attributed this to a catalytic effect of the non‐treated plastic surface on CsgA self‐assembly. This in turn suggested that CsgA TM and QM could form fibrils if provided with a nucleation point, prompting us to carry out seeding experiments by the addition of pre‐formed fibrils. Such experiments could also clarify whether the gatekeeper residues of TM and QM mainly inhibit the primary nucleation or elongation mechanism. The occurrence of fibril polymerization in the presence of fibril seeds would strongly indicate that the inhibiting effect of the gatekeeper residues is limited to fibril nucleation rather than elongation. All reactions were conducted with a constant monomeric concentration of 7.2 μM and preformed seed % was calculated based on the total protein mass. Self‐replicating processes such as secondary nucleation and fragmentation are virtually absent during the polymerization of CsgA (Andreasen et al., 2019). This means that relatively large (>1% of total protein mass) amounts of fibril seeds are required to promote fibrillation. Consistent with this, all CsgA variants required 1% preformed fibril seeds before any significant seeding effect was observed (Figure 3), allowing us to conclude that secondary processes such as elongation of existing fibrils are not major contributors to CsgA fibrillation. Since all 4 constructs can aggregate in the presence of seeds or a catalytic surface that likely mimics seeds in some capacity (Figure S1), the introduction of gatekeeper residues must affect fibril nucleation more than the elongation of existing fibrils.

Given that the mutations introduce Asp residues, it is possible that the inhibitory effect on fibrillation could be caused by the ionic repulsion of the acidic groups. If this was the case, such effects should be mitigated by shielding charges through increased ionic strength or by altering the pH. To address this, we fibrillated wt CsgA and CsgA DM in increasing concentrations of NaCl (Figure S2a) and at various pH values (Figure S2b). The addition of NaCl did not seem to have any restorative effects on CsgA DM, rather, it decreased the fibrillation propensity of both wt CsgA and CsgA DM (except for wt CsgA at 1000 mM NaCl). The pH study showed a different trend, with the amyloidogenicity of wt CsgA decreasing with pH from 9 to 3.3; CsgA DM displayed a peak fibrillation rate at pH 4.4–5.7, while the largest ThT signal was achieved at pH 7.4. These data suggest that a slightly protonated population of Asp residues in CsgA DM weakly promotes fibrillation while wt CsgA gains no benefits from a slightly acidic pH. Thus, simple electrostatic repulsion does not rationalize the effect of the gatekeeper residues.

In conclusion, the introduction of just two gatekeeper residues in R1 and R5 of CsgA significantly reduces the protein's propensity to form fibrils, while two additional gatekeeper residues completely inhibit the ability to form fibrils, mainly by inhibiting the primary nucleation processes unless fibrillation is stimulated by seeding or surface catalysis. However, we cannot distinguish between TM and QM effects.

### 3.2 wt CsgA, DM, TM, and QM all have the same fibrillar architecture

**FIGURE 4:**
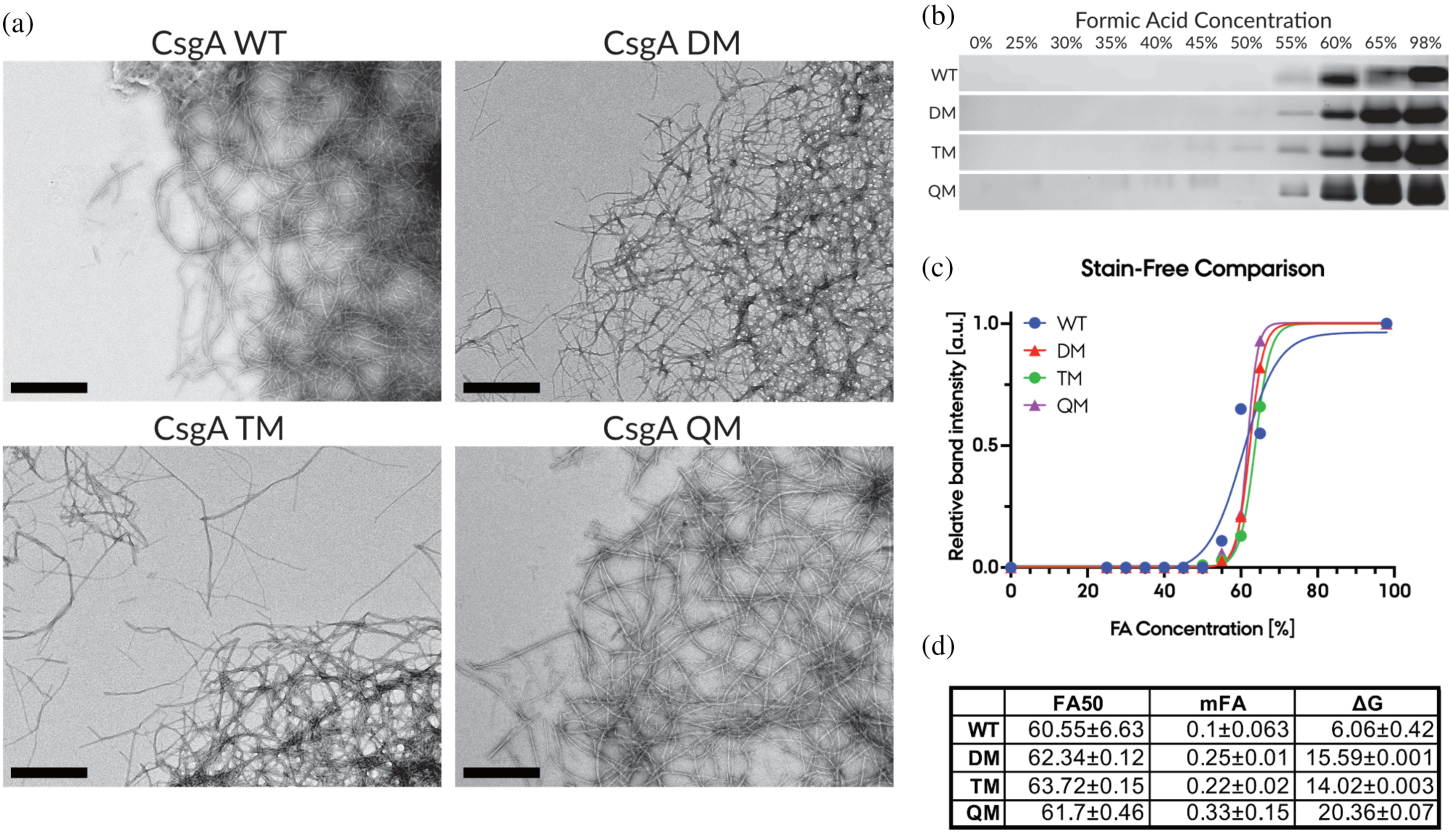
Despite different polymerization rates, all four CsgA variants appear visually similar and have the same association tendencies. The mature fibrils similarly have near identical stability towards formic acid dissolution. (a) Transmission electron microscopy (TEM) images of uranyl formate stained mature fibrils from the four CsgA variants (Scalebar, 500 nm). (b) SDS‐PAGE bands of samples taken from formic acid dissolution assay. Formic acid concentration is indicated in %‐V/V. All CsgA variants required 55% formic acid before dissolution was visible while the majority of the fibril mass was dissolved at 65% formic acid resulting in a strikingly similar FA50 value. (c) The intensity of each SDS‐PAGE band was measured and normalized to the intensity of the 98% band for each CsgA variant. The resulting data were plotted as a function of formic acid concentration and modeled with a formic acid dissolution model to determine FA50, mFA, and Δ*G* values. (d) FA50, mFA, and Δ*G* values determined from formic acid dissolution model.

Prompted by these very different aggregation properties, we used TEM to compare morphologies of mature fibrils, generated for all four CsgA variants by incubating monomers for 24 h in Eppendorf tubes at 37°C while shaking. The plastic surface of a standard Eppendorf tube allows both CsgA TM and QM to form fibrils, even in the absence of preformed seeds. When deposited on TEM grids, fibrils of all four variants were heavily associated laterally, forming thick cable‐like structures that ultimately led to large tangles of fibrils (Figure 4a). Isolated, free fibrils were rarely observed. Thus, once formed, the fibrils of CsgA DM, TM, and QM form fibrils that are indistinguishable from wt CsgA fibrils at TEM resolution. Nevertheless, it might be possible that the stability of these fibrils was affected. Sensitivity to protease treatment could in principle report on fibril stability but will do so in an indirect fashion. The compact structure of CsgA does not provide obvious points of proteolytic attack, so protein degradation will reflect a coupling between (usually slow) fibril dissociation and cleavage of the monomers. Rather, to measure fibril stability, we subjected the fibrils to increasing concentrations of formic acid, known to dissociate and dissolve functional amyloid at high concentrations in a quantitative manner (Christensen et al., 2020).

Dissolved monomeric CsgA was quantified by SDS‐PAGE (Figure 4b). Visual inspection of these gels showed very little difference in stability among the four CsgA variants. All underwent a transition from no solubilization <50% FA to complete solubilization >65% FA. Using densitometric quantification, data were fitted to a two‐state unfolding model (see M&M for details) to obtain the midpoint of denaturation [FA]^50%^, the free energy of unfolding Δ*G* and the dependence of Δ*G* on [FA] (*m*_FA_) (Figure 4c, summarized in Figure 4d). All four CsgA variants display very similar ${\left[\mathrm{FA}\right]}^{50\%}$ values and the three mutants also show very similar Δ*G* values. wt CsgA has a much lower Δ*G* value, but we attribute this to the large errors in the *m*_FA_ value due to noisy data in the transition region.

We conclude that despite a very high kinetic barrier to fibril formation, the three mutants all show the same morphology and stability as wt CsgA. Thus, the gatekeeper residues in CsgA primarily affect the initial formation of fibrils, likely by discouraging intramolecular or intermolecular interactions that are normally required to obtain the correct folding for fibril formation.

### 3.3 CHAPS interacts differently with wt CsgA, DM, TM, and QM

We decided to probe whether this early‐stage blockage of fibrillation made the mutants more sensitive to other modulators of fibrillation. Harsh anionic surfactants such as SDS inhibit the fibrillation of functional amyloid at high concentrations (Najarzadeh et al., 2019). We therefore turned to the gentler zwitterionic surfactant CHAPS which only had modest effects on wt CsgA, even at concentrations well above its critical micelle concentration which we determined to be around 3.2 mM using pyrene fluorescence (Figure S3).

**FIGURE 5:**
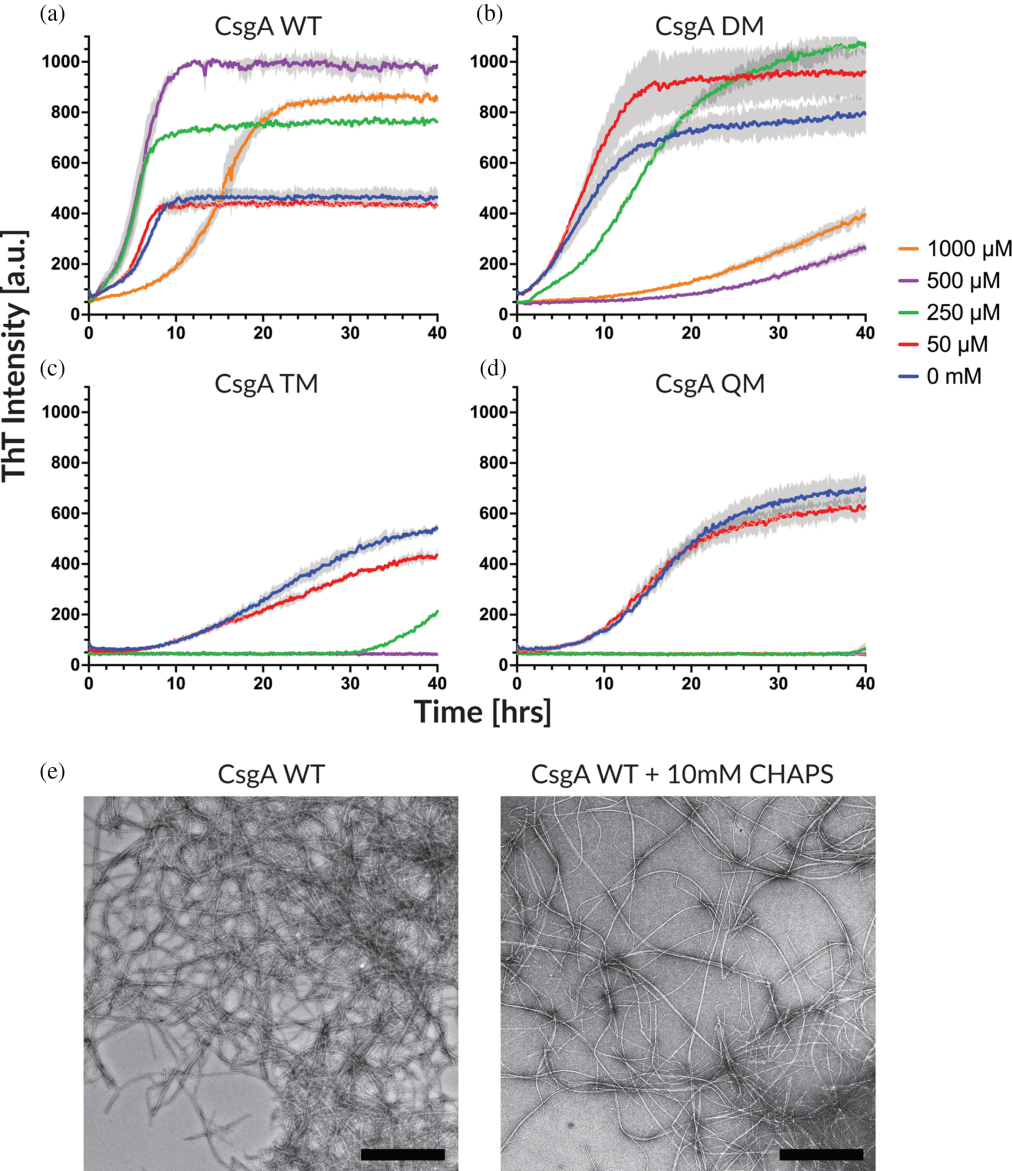
The mild detergent CHAPS interacts with wt CsgA and DM to produce distinct transitions in the polymerization profile and produces fibrils with a reduced tendency to form clumps. All ThT aggregation assays were performed in non‐treated polystyrene 96‐well plates. (a) ThT fluorescence traces of 7.4 μM wt CsgA polymerization in the presence of CHAPS. Distinct transitions in the polymerization profile happen at 250 and 1000 μM CHAPS. (b) ThT fluorescence traces of 7.4 μM CsgA DM polymerization in the presence of CHAPS. The same distinct transitions are present both occur at 50 and 250 μM instead. (c, d) ThT fluorescence traces of 7.4 μM CsgA TM and QM polymerization in the presence of CHAPS. Only one transition is visible at 50 μM. A small but significant difference in lag phase is visible between TM and QM at 250 μM CHAPS, suggesting QM to be the most inhibited CsgA variant. (e) TEM images of wt CsgA fibril formed in the absence (left) and presence (right) of 10 mM CHAPS. The presence of CHAPS during the fibrillation process produces mature fibrils that are significantly more disperse compared with wt CsgA without CHAPS (Scalebar, 500 nm).

In 50 μM CHAPS, there was no effect on wt CsgA fibrillation (Figure 5a). 250–500 μM CHAPS led to an increased primary nucleation rate as well as an increase in ThT‐intensity (Figure 5a), while ≥1 mM CHAPS mM again saw a shift in the aggregation kinetics, with a drastic decrease in primary nucleation and elongation rate (Figures 5a and S4). At and above 10 mM CHAPS, the aggregation rate for wt CsgA steadily declined with CHAPS concentration until we reached a plateau at 150 mM (Figure S4). We saw no evidence of direct contact between CHAPS and CsgA since the critical micelle concentration (CMC) was only insignificantly affected by CsgA, rising from 3.2 to 3.4 mM in the absence and presence of CHAPS, respectively (Figure S3).

We hypothesized that the mild perturbation of the aggregation mechanism by CHAPS might resolve the effect of the fourth gatekeeper residue introduced in QM. We monitored the fibrillation of all four CsgA variants in the presence of 0–1 mM CHAPS, using non‐coated 96‐well plates to promote fibril formation of CsgA TM and QM (Figure 5a–d). CsgA DM responded to CHAPS with the same three concentration regimes as observed for wt CsgA but at much lower CHAPS concentrations. Thus, fibrillation of CsgA DM was almost completely inhibited already at 500 μM CHAPS, an effect only observed at 150 mM CHAPS for wt CsgA. This suggests that DM fibrillation is so compromised compared to wt CsgA that only small amounts of a mild surfactant are sufficient to severely reduce aggregation. This effect is even more pronounced for mutants TM and QM, for which there was no increase in fibrillation rate at the lowest CHAPS concentrations but simply a large increase in lag‐phase already at 250 μM CHAPS. TM and QM show lag times of 30 and 40 h, respectively, at 250 μM CHAPS, showing that CsgA QM is slightly more inhibited by the additional gatekeeper residue but with the largest effects caused by the first three gatekeeper residues. At 500 μM CHAPS or above, both TM and QM were completely inhibited from aggregating. In principle, CHAPS could work indirectly on aggregation through effects on surface tension; however, CHAPS' relatively high cmc means that there is only a modest decline in surface tension at sub‐mM concentrations (cf. the observed decline in surface tension by only ~8 mN/m up to the CMC in figure 1 of Lunkenheimer et al., 2007).

These results prompted us to investigate if the interaction between CHAPS and CsgA affected the morphology of the mature fibrils. TEM images of CsgA fibrils grown in the absence and presence of 10 mM CHAPS showed that CHAPS led to much more dispersed fibrils and much less dense clumps of fibrils (Figure 5e) than in its absence. This allowed for clear imaging of both individual fibers and fiber bundles, suggesting that CHAPS not only interferes with the initial fibril formation mechanism but also coats the fibrils, reducing the otherwise strong lateral association. This data likewise demonstrates how CsgA fibrils consist of an ensemble of fibers that are formed from varying amounts of intertwined fibrils (Figure S5). Our observations are nicely consistent with the very recent report by Liu and coworkers who used the nonionic surfactant Tween 20 to provide better dispersion of CsgA fibrils, leading to a 3.62 Å cryoEM structure of CsgA in combination with AlphaFold (Bu et al., 2024), in extension of the pioneering work by Remaut and coworkers (Sleutel et al., 2023).

### 3.4 Growth measurements of single fibrils confirm a significant slowing of the elongation rate of CsgA DM

Having investigated the growth parameters of CsgA and the gatekeeper mutants using ThT fluorescence bulk measurements, we set out to measure the growth behavior of individual fibrils using total internal reflection fluorescence (TIRF) microscopy, which allows us to track individual fibrils.

As opposed to most other TIRF amyloid studies that use amyloid‐specific fluorescence probes such as Nile Blue or ThT, we opted to use an AlexaFluor 488 cross‐linked fluorescent dye to get greater contrast in our measurements. Only wt CsgA and CsgA DM were recorded since these were the only two CsgA variants that were able to form fibrils in the absence of seeds or catalytic agents. Both growth experiments were conducted at 1 μM monomeric concentration to avoid crowding at the imaging surface.

**FIGURE 6:**
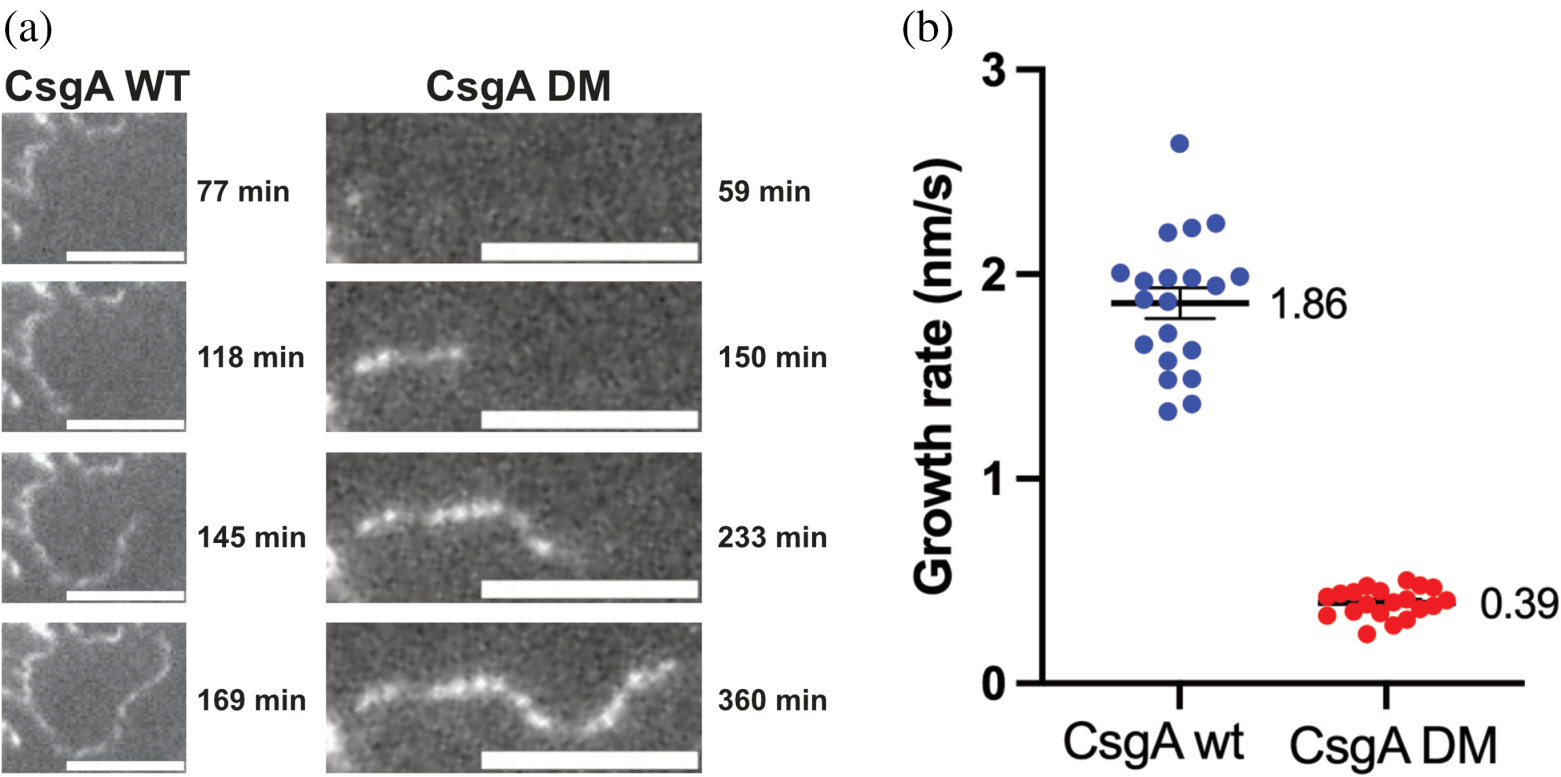
TIRF microscopy allows accurate determination of growth rate of individual fibrils for wt CsgA and CsgA DM. (a) Example of time series of growth of wt CsgA and CsgA DM fibrils, showing real‐time tracking of individual fibrils, giving accurate growth rates determined on a single‐fibril level. Scale bare is 5 μm. (b) Growth rate determined for 20 wt CsgA fibrils (blue) with an average growth rate of 1.86 nm/s and 20 CsgA DM fibrils (red) with an average growth rate of 0.39 nm/s. Error bars indicate 95% standard error of mean (SEM).

The growth rate of the fibrils captured by the TIRF microscope was determined simply by measuring the length of the fibrils over time with ImageJ and then dividing it by the length of time in which the fibrils were growing (Figure 6a). End of growth was defined as either the point in time when the fibril would no longer grow or when the measurements were stopped. The growth rates of 20 fibrils were measured for both wt CsgA and CsgA DM (Figure 6b). With a growth rate of 1.86 nm/s, wt CsgA fibrils elongate almost 5 times faster than CsgA DM at 0.39 nm/s.

The results are highly congruent with the observations made in the bulk ThT measurements with wt CsgA showing a significantly higher fibrillation rate compared with CsgA DM. This direct observation of the growth rate further demonstrates that the introduction of gatekeeper residues has a large impact on the elongation rate of CsgA.

### 3.5 NMR spectroscopy shows that the monomeric structures of CsgA variants are intrinsically disordered and insensitive to mutation

**FIGURE 7:**
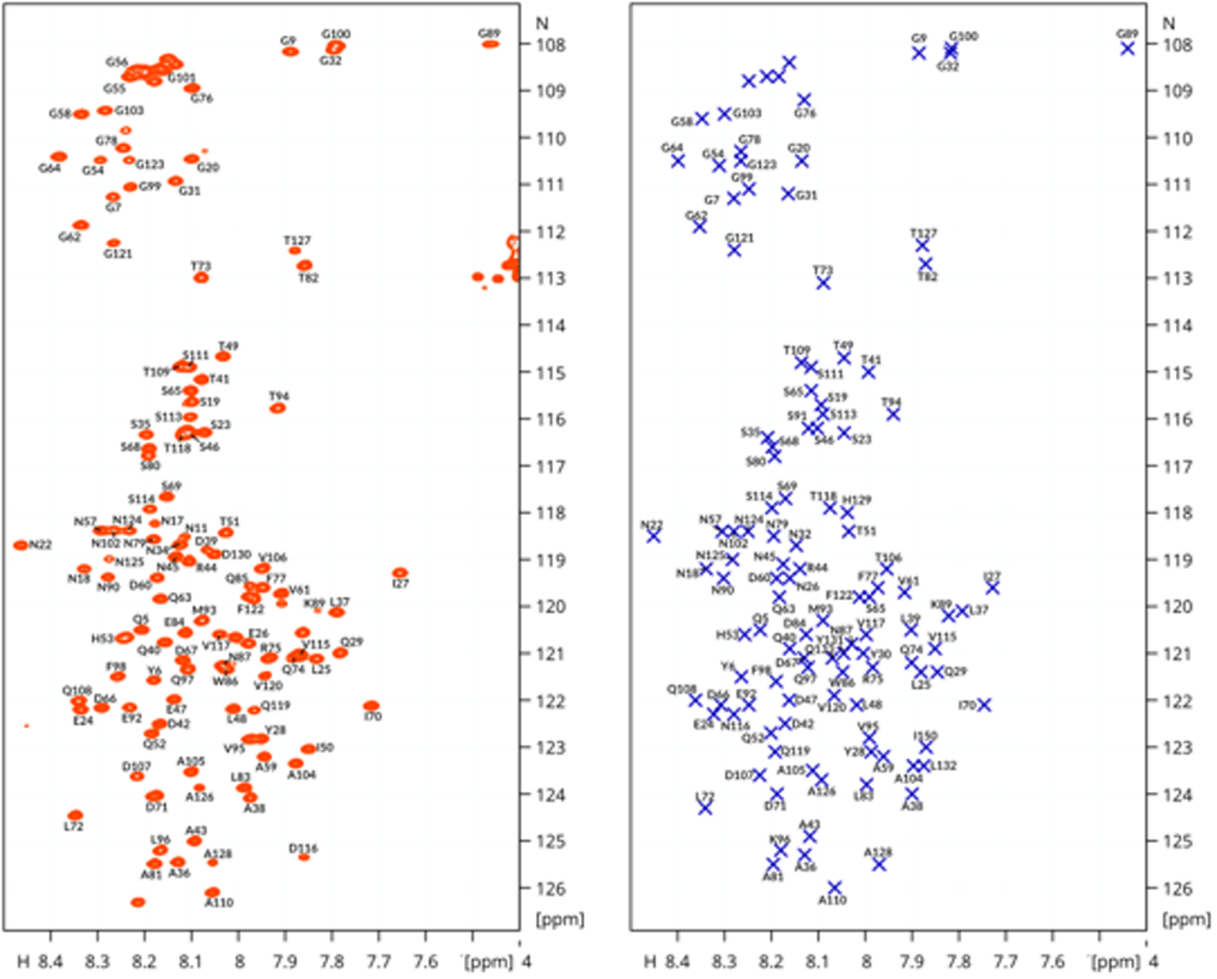
^15^N–^1^H HSQC spectrum of 250 μM CsgA QM in 20 mM Na_2_HPO_4_, pH 7.2, 10°C recorded at 950 MHz (red peaks, this study) and wt CsgA (blue crosses, from Sewell et al., 2020). Note that wt CsgA assignments are numbered according to the full‐length CsgA sequence, including the 20‐residue signal peptide, while our assignments are based on mature CsgA.

A major obstacle when conducting experiments on any amyloidogenic protein is their inevitable aggregation, especially when experiment conditions require high concentrations. We reasoned that the far less amylodogenic CsgA QM variant would therefore be advantageous to use for this purpose. Sewell and coworkers have previously recorded and assigned a Heteronuclear Single Quantum Coherence (HSQC) spectrum of wt CsgA (Sewell et al., 2020) and the spectrum of QM was highly similar, except for the few positions in the immediate vicinity of the amino acid substitutions. We recorded high‐resolution 3D Nuclear Overhauser Effect Spectroscopy ^15^N (NOESY)‐HSQC and 3D Total Correlation Spectroscopy ^15^N (TOCSY)‐HSQC spectra, and found that the CsgA QM spectra aligned sufficiently well with wildtype to transfer literature assignments when corroborated by connectivities in the 3D NOESY and TOCSY spectra. ^1^H–^15^N correlations were assigned for 110/131 (84%) residues (excluding the C‐terminal His_6_‐tag). Many of the unassigned peaks are located in the Gly region of the spectrum or are part of multiple overlapping peaks, making the assignment ambiguous. Figure 7 shows HSQC spectra of wt and QM side‐by‐side. To demonstrate that the structural ensemble was unaltered, we compared the experimentally obtained assignments with random coil predictions for the respective sequences using the POTENCI standard (Nielsen & Mulder, 2018) to obtain secondary chemical shifts (Δδ = δ_exp_ − δ_POTENCI_). Figure S6 shows these results and established that the conformational ensembles are not detectably altered by the gatekeeper mutations.

### 3.6 Gatekeeping residues affect the formation of transiently bound states

While Cryo‐EM has emerged as the leading technique for solving the structure of mature fibrils, information on intermediate species and the disordered state is still required to elucidate the mechanism of fibril formation. Nuclear magnetic resonance (NMR) remains the preferred technique for such experiments and has previously been used to probe the structure of CsgA both in its fibril and disordered state (Sewell et al., 2020; Shewmaker et al., 2009). Dark‐state Exchange Saturation Transfer (DEST) serves as a powerful tool in solution NMR studies, allowing us to characterize interactions between an “NMR‐visible” species and an “NMR‐invisible” species transiently bound to a massive (>1 MDa) macromolecule. The bound state experiences significantly slower tumbling, making it invisible to traditional solution NMR methods. DEST leverages this difference in motional freedom to provide valuable insights into such interactions. Here, we employed DEST to probe the transient binding of monomeric CsgA proteins to their corresponding fibrils (Fawzi et al., 2012). Based on the findings in the previously described experiments, we hypothesized that the introduced gatekeeper residues would decrease the monomer‐fibril association and would correlate with the number of gatekeepers.

Since the high molecular weight of fibrils leads to rapid decay of their transverse magnetization due to large *R*_2_ (transverse relaxation) rates, they remain invisible in NMR spectra in contrast to the freely tumbling monomers. Consequently, as monomers associate and dissociate with fibrils in the solution, their *R*_2_ rates increase, resulting in an overall increase in the observed *R*_2_ rate of the CsgA monomers. The magnitude of this transverse relaxation rate increase in the presence of fibrils directly reflects the association rate between monomers and fibrils (Fawzi et al., 2010). In the first experiment, we allowed monomeric wt CsgA to form fibrils over the course of 4 days, measuring the signal decay from fibril formation (Figure S7a). After 4 days of fibrillation, the transverse relaxation rate of the remaining CsgA monomers was measured as 13.4 ± 0.4 s^−1^ (Figure S7b). During the 4‐day fibrillation process, the transverse relaxation rate was likewise measured at various time points and no change in the relaxation rate between these time points was observed, indicating that fibrils had formed from the start of the experiment, producing a dark state through the interaction with monomeric CsgA. To probe the change in relaxation rate induced by fibril‐monomer association, a sample with little to no interaction between fibrils and monomers was required. The previous experiment indicated that wt CsgA produces small fibrils as soon as it has been buffer changed from GnHCl solution and was thus not suitable. Instead, a similar experiment was performed using CsgA QM, and signal decay and transverse relaxation rate were followed over a 4‐day period (Figure S7a). Similar to the wt CsgA measurements, the transverse relaxation rate did not change over the course of the experiment but no loss in signal was detected, indicating that no fibrils had formed.

We hypothesized that the transverse relaxation rate of CsgA QM was unaffected by monomer‐fibril interactions. To confirm this hypothesis, we measured the *R*_2_ rate of CsgA QM in the presence of 5% pre‐formed fibrils and observed no difference in the *R*_2_ rate (Figure S7c). The final measurement of the CsgA QM sample produced a transverse relaxation rate of 9.1 ± 0.2 s^−1^ (Figure S6B). Assuming that the *R*_1_ relaxation (3 s^−1^) and *R*_2_ relaxation (9.1 s^−1^; Figure S7b) of CsgA QM accurately represent all monomeric CsgA proteins in solution, and further assuming that cross‐relaxation is significant in the fibril‐bound state (Fawzi et al., 2010), an estimate of the apparent first‐order association rate constant *k*_on_^app^ could be calculated by simply subtracting the *R*_2_ rate of the CsgA QM experiment from wt CsgA experiment, yielding a value of *k*_on_^app^ = 4.3 s^−1^, indicating significant exchange between monomers and fibrils of wt CsgA.

**FIGURE 8:**
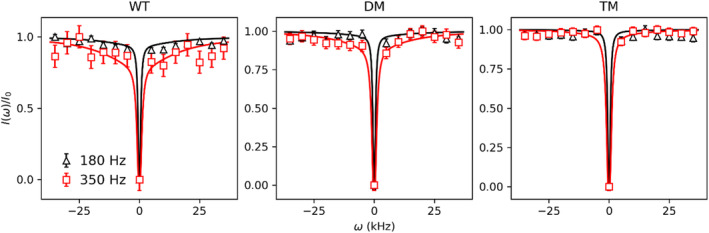
Attenuation (*I*(ω)/*I*_0_) of the integrated intensity of monomeric CsgA proteins by transfer of saturation from the transient fibril‐bound (“dark”) state following application of off‐resonance radio frequency (ω) fields as a function of offset from the water resonance, at field strengths of 180 Hz (black triangles) and 350 Hz (red squares). The data from both field strengths were simultaneously fit to a two‐site exchange model using the Bloch‐McConnell equations to obtain dissociation rates reflecting the population of CsgA in the transiently bound state.

Similar experiments were conducted using CsgA DM and TM monomers (CsgA TM required the addition of 5% pre‐formed fibrils due to slow fibrillation), and the resulting observed saturation profiles (Figure 8) were fit using *R*_2_^dark^ = 31,000 ± 3000 s^−1^ and the values provided in Table 1. The k_on_^app^ was assumed to be the same for all CsgA proteins. The results show a clear trend: the fraction of protein in the transiently bound state (calculated from the *k*_off_/*k*_on_^app^ ratio) significantly decreases with the increasing number of gatekeeping residues in the DM and TM mutants, while QM showed no transiently bound state at all.

**TABLE 1:** Fitted parameters for the observed saturation profiles in Figure 8.

Protein	*k*_off_ (s^−1^)	% of protein in transiently bound state
wt	900 ± 140	0.5
DM	1900 ± 400	0.2
TM	7000 ± 4000	0.01

## 4 DISCUSSION

Gatekeeper residues (e.g., charged and β‐breaker residues) have long been known to play an important role in suppressing misfolding of globular proteins by destabilizing alternative folded states such as amyloid (Beerten, Jonckheere, et al., 2012; Ganesan et al., 2016; Otzen et al., 2000; Rousseau et al., 2006). Gatekeeper residues are likewise found in functional amyloid proteins, where they seem to play an analogous role, delaying aggregation until the protein reaches its destination. wt CsgA contains gatekeeper residues in repeats R2–R4, whose removal by mutation significantly accelerated fibrillation (Wang et al., 2010). In the present study, we set out to determine how the fibrillation mechanism would change when new Asp gatekeeper residues were introduced into R1 and R5. The gatekeepers were introduced in a stepwise fashion to investigate how each contributed to the inhibition of the fibril formation and if their effect was additive. The introduction of two Asp residues in CsgA DM resulted in a significant reduction in primary nucleation and elongation rates, while fibrillation was completely inhibited for CsgA TM and CsgA QM. Thus, the introduction of multiple copies of a single type of mutation (X → Asp) was sufficient to completely eliminate spontaneous fibrillation of CsgA. Interestingly, CsgA TM and CsgA QM were all able to form fibrils in the presence of preformed seeds or a catalytic surface, suggesting that the introduced gatekeeper residues mainly affected fibril nucleation and elongation only to a smaller degree. As the HSQC NMR spectra are virtually unchanged by the introduction of gatekeeper residues, and the analysis of secondary chemical shifts did not show significant differences, this data suggests that the productive states for nucleation are only present at minute amounts at equilibrium. Changes in concentration and electrostatics (pH, ionic strength) may strongly modulate self‐association, albeit with undetectable signatures on the monomeric species. The small populations of these productive species cause the aggregation process to be slow, while at the same time highly susceptible to its milieu (*vide infra*).

### 4.1 Single‐fibril tracking by TIRF microscopy directly illustrates slower elongation rates of gatekeeper mutants

TIRF microscopy (TIRFm) has traditionally been used to characterize fibril structure, amyloid branching, and growth fingerprinting (Andersen et al., 2009). Recent advances in machine learning for data analysis have sparked new interest in the tracking of fibrillar structures (Bender et al., 2024). To probe the degree of elongation inhibition, we utilized TIRFm to track the growth of individual wt CsgA and CsgA DM fibrils. Since the CsgA DM elongation rate is almost 5 times slower than that of wt CsgA, this result highlighted specifically how elongation is affected by the introduction of gatekeeper residues. The experiment likewise serves as an example of how TIRFm can be utilized as a powerful tool for amyloid fibril tracking and growth measurement on the single‐particle level. Using basic image manipulation tools, a robust measurement of fibril growth rate can be obtained in a very short time span.

### 4.2 Gatekeeper residues tune fibrillation kinetics but not fibrillation stability

Aggregation in the presence of the mild detergent CHAPS showed a small but significant increase in the lag phase of CsgA QM compared to TM and DM. Thus, each new Asp added to CsgA increases the barrier to fibrillation. This clear correlation between the number of introduced gatekeeper residues and reduced amyloidogenicity suggests that evolution can utilize the number of gatekeeper residues to fine‐tune the amyloidogenicity of functional amyloids and the stability of aggregation‐prone regions in globular proteins. For CsgA, these results strongly suggest that the number and placement of native gatekeeper residues are optimized so that the protein remains disordered for the time required to reach the nucleator protein CsgB on the outer membrane of the cell. This ensures correct and controlled aggregation and minimizes the otherwise toxic effects of the runaway‐aggregation.

Surprisingly TEM imaging demonstrated that mature fibrils from all four CsgA proteins, despite their different aggregation kinetics, were visually indistinguishable. Not only were the fibrils visually similar but they also had near‐identical stability towards formic acid dissolution, suggesting that once formed, the fibrils retain both structure and stability. This further supports the idea that gatekeeper residues mainly affect the nucleation phase of the aggregation mechanism while preserving the amyloid fold. From an evolutionary point of view, this behavior seems highly beneficial to organisms with functional amyloids since it preserves the function of the amyloid fold but delays or even eliminates aggregation until initiated by external nucleators.

### 4.3 DEST NMR studies show a reduction in the degree of transient binding to fibrils caused by gatekeeper residues

**FIGURE 9:**
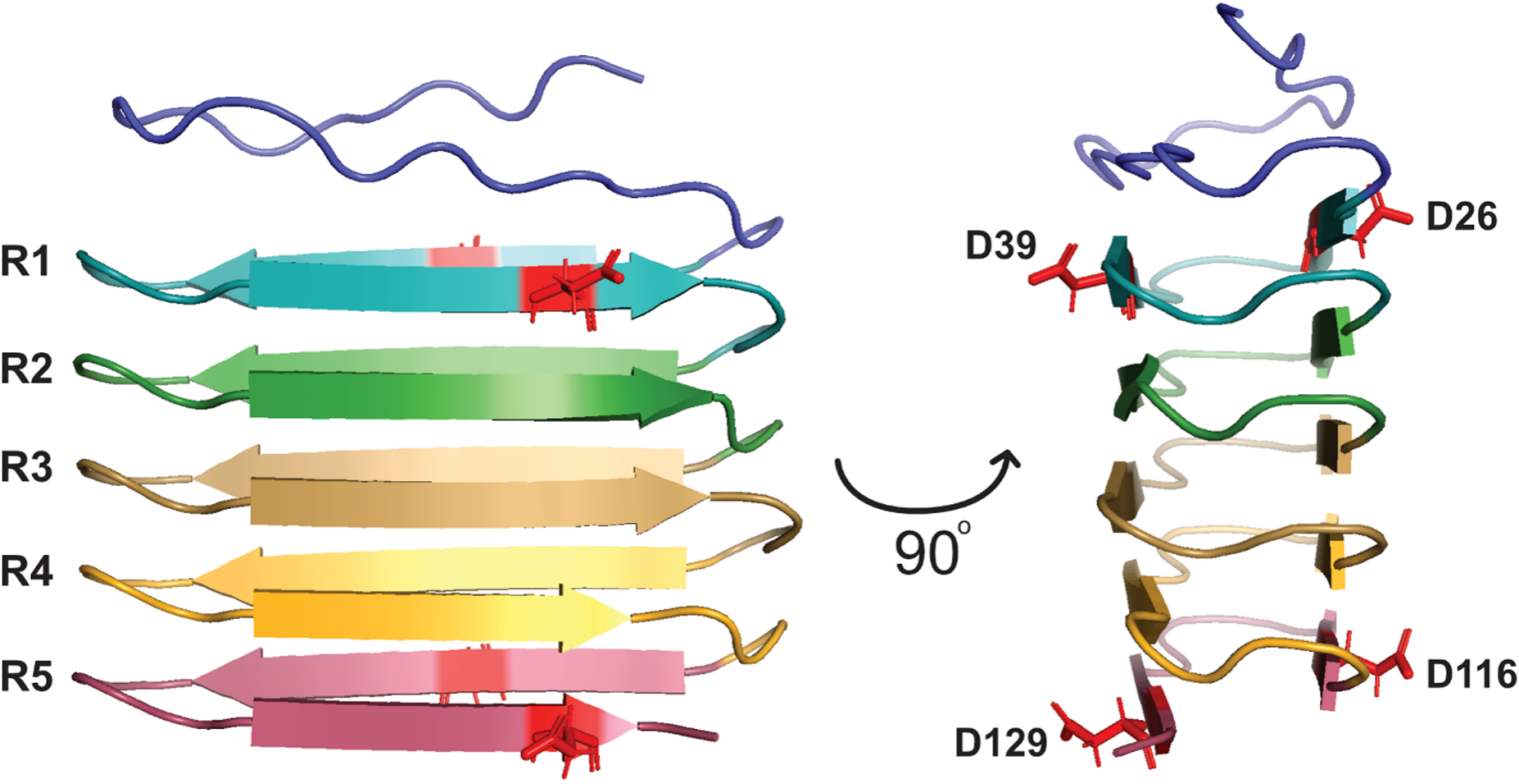
AlphaFold structure of CsgA QM shows that it adopts a β‐hairpin very similar to wt CsgA with five repeats. The four Asp gatekeepers residues introduced in CsgA QM (red) all point outwards from the amyloid core. This might explain their lack of influence on the stability and structure of the mature fibrils while still affecting initial nucleation. For clarity, the His‐tag has been removed.

An analysis of the AlphaFold model of CsgA QM found that all four introduced gatekeeper residues point outwards from the amyloid core (Figure 9). This could explain their minimal effect on fibril structure and stability while still affecting nucleation. It is likely that the association between two or more monomers during fibril nucleation is weakened by electrostatic repulsion from the additional Asp residues. This is further supported by a significant decrease in the population of transiently bound states observed in the DM and TM mutants compared to the wild‐type protein through DEST experiments (Table 1). The DEST method has previously been used to observe the transiently bound states of Aβ and α‐synuclein (Bodner et al., 2009), and transient interactions have been documented as crucial early steps in the nucleation process (Fawzi et al., 2010; Karamanos et al., 2014). Given the observed decrease in CsgA's transiently bound population with gatekeeper mutations, we propose that these interactions play a role in the nucleation and/or growth of CsgA fibrils. While the DEST experiments do not provide any information on the location of the interactions, the monomers could either be associating with the fibril surface or the growing ends of fibrils; it seems clear that prolonged monomer‐fibril association is crucial to fibril nucleation and/or fibril growth. The introduction of additional charges from the gatekeeper residues in the CsgA mutants may be the cause of the disruption of this interaction, ultimately resulting in lower amyloidogenicity.

The DEST experiments also highlight the potential of using CsgA QM as a substitute for wt CsgA in prolonged NMR experiments that are otherwise complicated by rapid fibril formation at elevated concentrations. CsgA QM could be an excellent candidate for studies of the monomeric structure, its interactions with the remaining curli proteins, and even structural analysis of the early oligomeric species that may be formed prior to nucleation. Additionally, the use of gatekeeper residues could also be an invaluable tool in the development of nanomaterials based on amyloid fibrils (Das et al., 2018; Gilbert & Ellis, 2019; Hauser et al., 2014; Mankar et al., 2011) in situations where fibrillation kinetics but not stability need to be modulated.

## AUTHOR CONTRIBUTIONS

**William P. Olsen:** Conceptualization; investigation; formal analysis; writing – original draft; writing – review and editing; visualization. **Gaston Courtade:** Investigation; formal analysis; writing – review and editing. **Samuel Peña‐Díaz:** Conceptualization; investigation. **Madhu Nagaraj:** Conceptualization; investigation. **Thorbjørn V. Sønderby:** Conceptualization; investigation. **Frans A. A. Mulder:** Conceptualization; formal analysis; writing – review and editing. **Mette G. Malle:** Conceptualization; investigation; writing – review and editing. **Daniel E. Otzen:** Conceptualization; funding acquisition; project administration; writing – original draft; writing – review and editing.

## CONFLICT OF INTEREST STATEMENT

The authors declare no conflict of interest.

## Supporting information


**DATA S1.** Supporting Information.
