# Supplementary material for: CsgA gatekeeper residues control nucleation but not stability of functional amyloid
Source: Protein Sci. 2024 Sep 20;33(10):e5178. doi: 10.1002/pro.5178 (PMC11414021; doi:10.1002/pro.5178)
Supplement: Supplementary file 1 — DATA S1. Supporting Information. [file PRO-33-e5178-s001.pdf]

Supplementary information

William P. Olsen[1,2], Gaston Courtade[3], Samuel Peña-Díaz[1], Madhu Nagaraj[1], Thorbjørn V. Sønderby[1], Frans A. A. Mulder[1,4,5], Mette G. Malle[1], Daniel E. Otzen[1]

[1] Interdisciplinary Nanoscience Center (iNANO), Aarhus University, Aarhus C, Denmark

[2] Sino-Danish College (SDC), University of Chinese Academy of Sciences, Beijing, China

[3] Norwegian Biopolymer Laboratory (NOBIPOL), Department of Biotechnology and Food Science, NTNU Norwegian University of Science and Technology, Trondheim, Norway

[4] Johannes Kepler University, Linz, Austria

[5] Institute for Chemistry, Aarhus University, Aarhus C, Denmark

PROTEIN SEQUENCES

>CsgA WT
MGVVPQYGGGGNHGGGGNNSGPNSELNIYQYGGGNSALALQTDARNSDLTITQHGGG
NGADVGQGSDDSSIDLTQRGFGNSATLDQWNGKNSEMTVKQFGGGNGAAVDQTASNSS
VNVTQVGFGNNATAHQYHHHHHH

>CsgA DM
MGVVPQYGGGGNHGGGGNNSGPNSELDIYQYGGGNSALALQTDARNSDLTITQHGGG
NGADVGQGSDDSSIDLTQRGFGNSATLDQWNGKNSEMTVKQFGGGNGAAVDQTASNSS
VNVTQVGFGNNATADQYHHHHHH

>CsgA TM
MGVVPQYGGGGNHGGGGNNSGPNSELDIYQYGGGNSALALQTDARNSDLTITQHGGG
NGADVGQGSDDSSIDLTQRGFGNSATLDQWNGKNSEMTVKQFGGGNGAAVDQTASNSS
VDVTQVGFGNNATADQYHHHHHH

>CsgA QM
MGVVPQYGGGGNHGGGGNNSGPNSELDIYQYGGGNSALADQTDARNSDLTITQHGGG
NGADVGQGSDDSSIDLTQRGFGNSATLDQWNGKNSEMTVKQFGGGNGAAVDQTASNSS
VDVTQVGFGNNATADQYHHHHHH

Figure S1

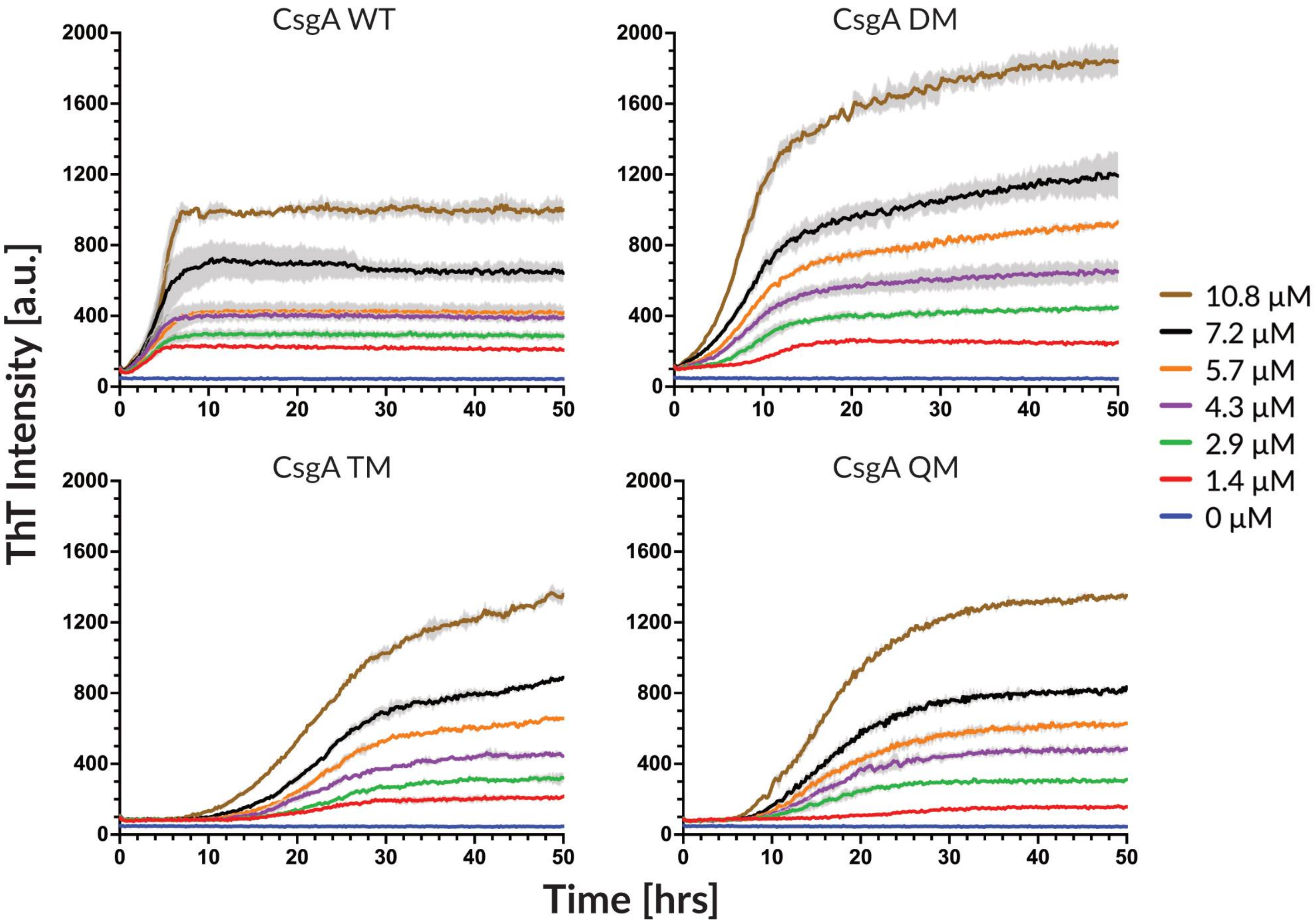

Fig. S1: All CsgA variants experience surface-catalysis in non-treated polystyrene 96-well plates. The effect is best seen for CsgA TM and QM, of which both readily form fibrils in these plates while wt CsgA and DM have significantly decreased lag-phase. ThT fluorescence trace of fibrillation of wt CsgA, CsgA DM, CsgA TM, and CsgA QM. Error bars indicate 95% standard error of mean (SEM) with 3 technical repliates of each sample.

Figure S2

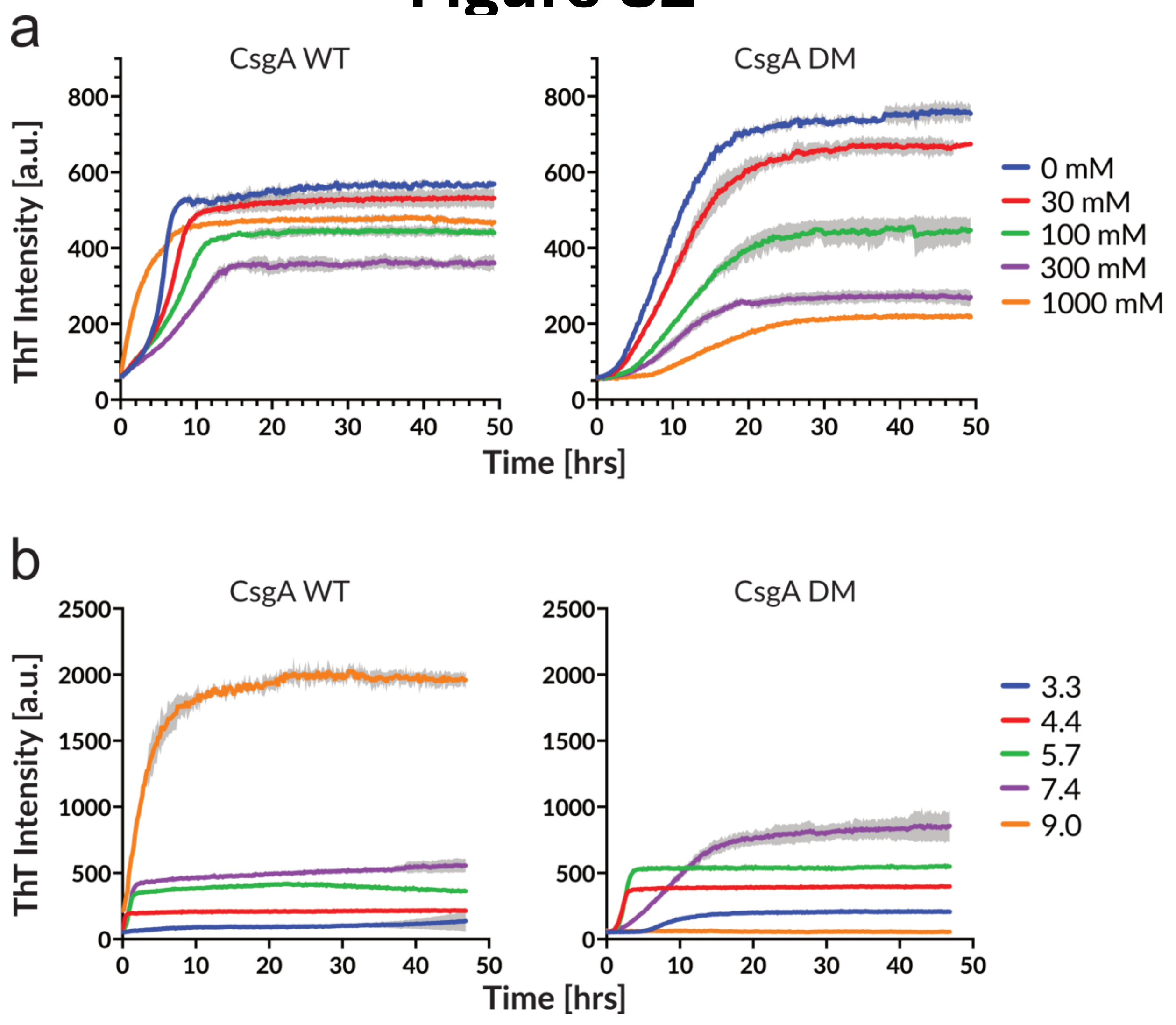

Fig. S2: (a) ThT fluorescence trace of fibrillation of wt CsgA and CsgA DM in the presence of 0-1000mM NaCl. The addition of salt decreases amylogenecity of both wt CsgA and CsgA DM, except for wt CsgA at 1000mM NaCl. (b) ThT fluorescence trace of fibrillation of wt CsgA and CsgA DM at various pH values between 3.3 and 9.0. wt CsgA experiences the highest amylogenecity at pH 9 while CsgA DM experiences the highest fibrillation rate at 5.7.

Figure S3

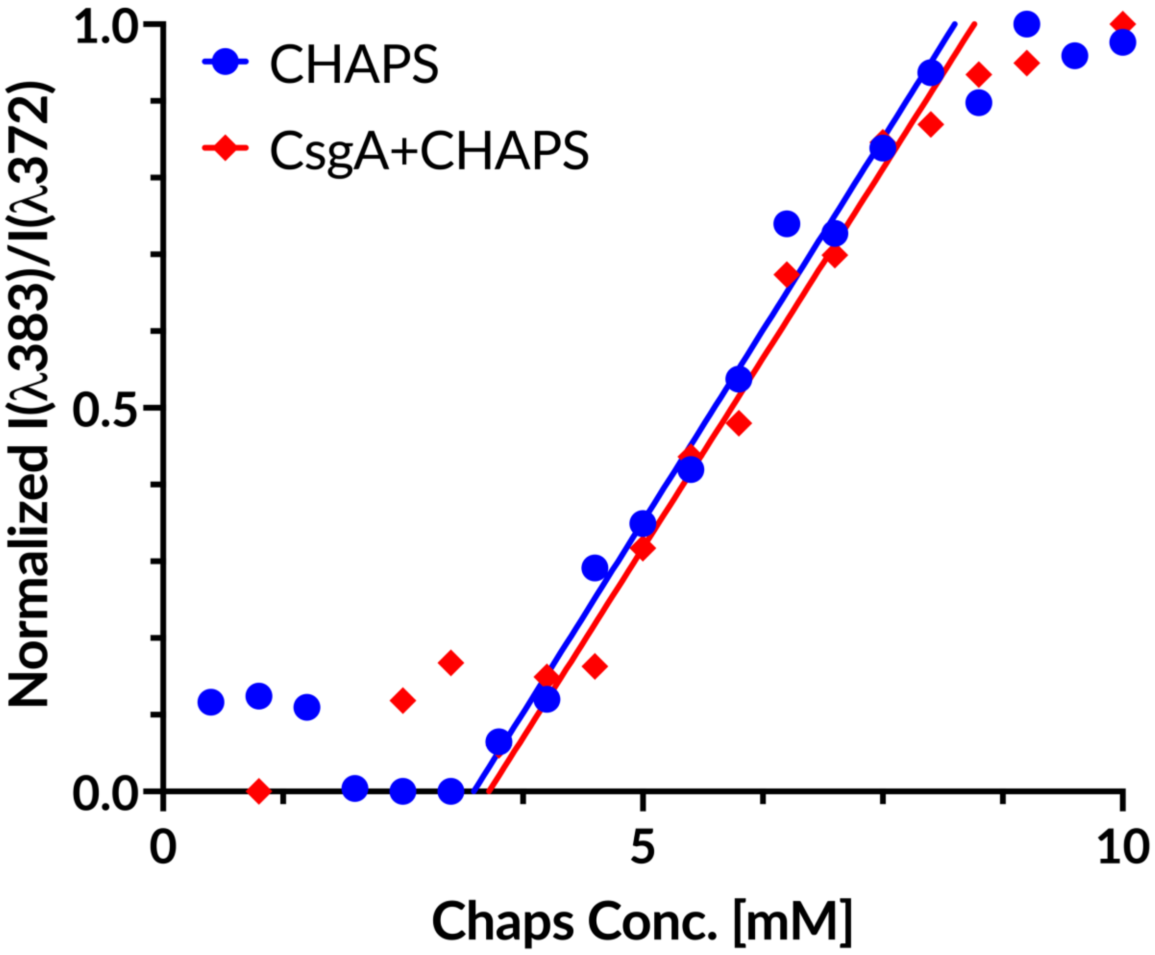

Fig. S3: The CMC of CHAPS in the absence and presence of 15 µM monomeric wt CsgA was determined by changes in the absorption of pyrene using CD spectroscopy. The presence of CsgA did not influence micelle formation.

Figure S4

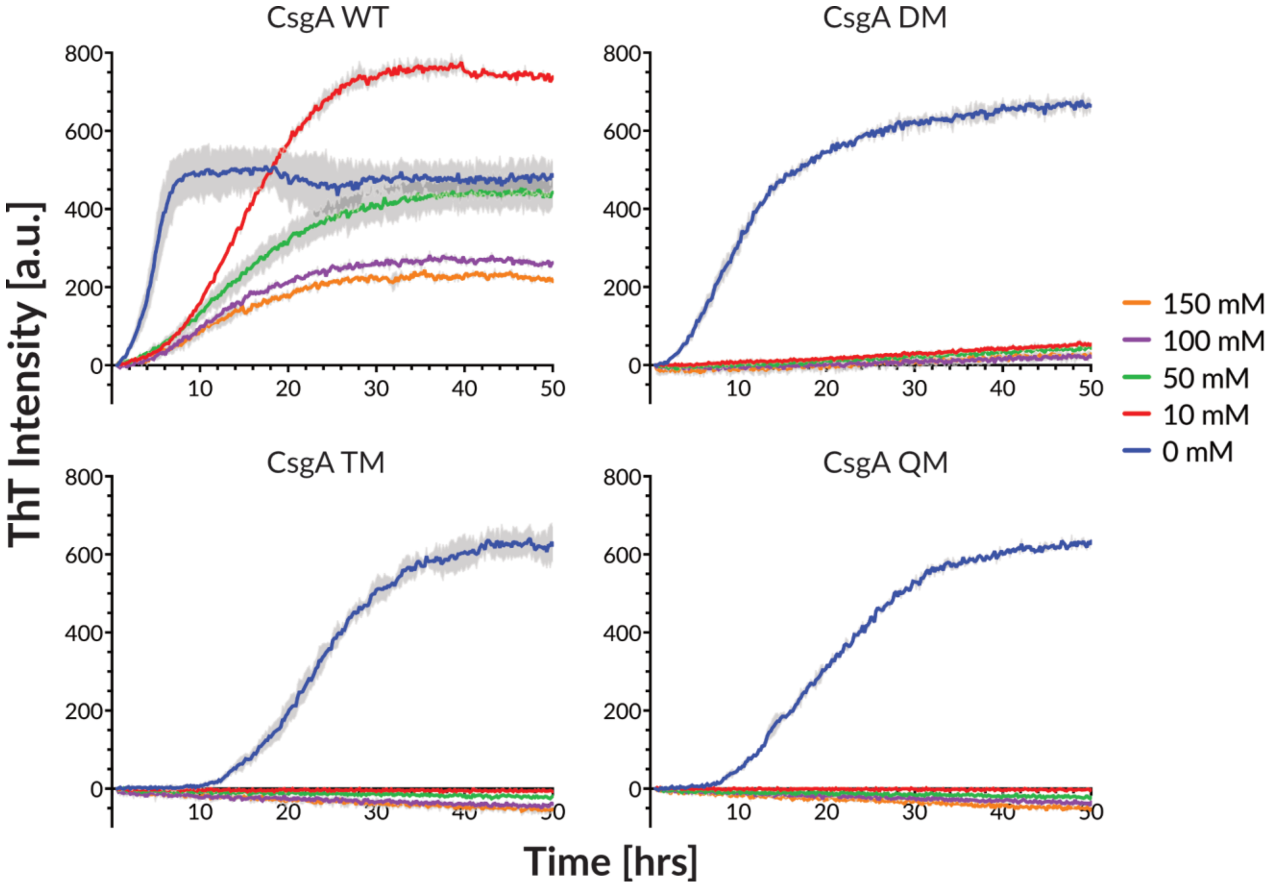

Fig. S4: CHAPS have mostly inhibitory effects when used at or above 10 mM for all CsgA variants. wt CsgA remain capable of polymerization albeit to a lesser degree even at 150 mM while DM, TM and QM are all completely inhibited. ThT fluorescence traces of 7.4 µM wt CsgA, DM, TM, and QM polymerization in the presence of 10-150 mM CHAPS. All experiments were performed in non-treated polystyrene 96-well plates. Error bars indicate 95% standard error of mean (SEM) with 3 technical repliates of each sample.

Figure S5

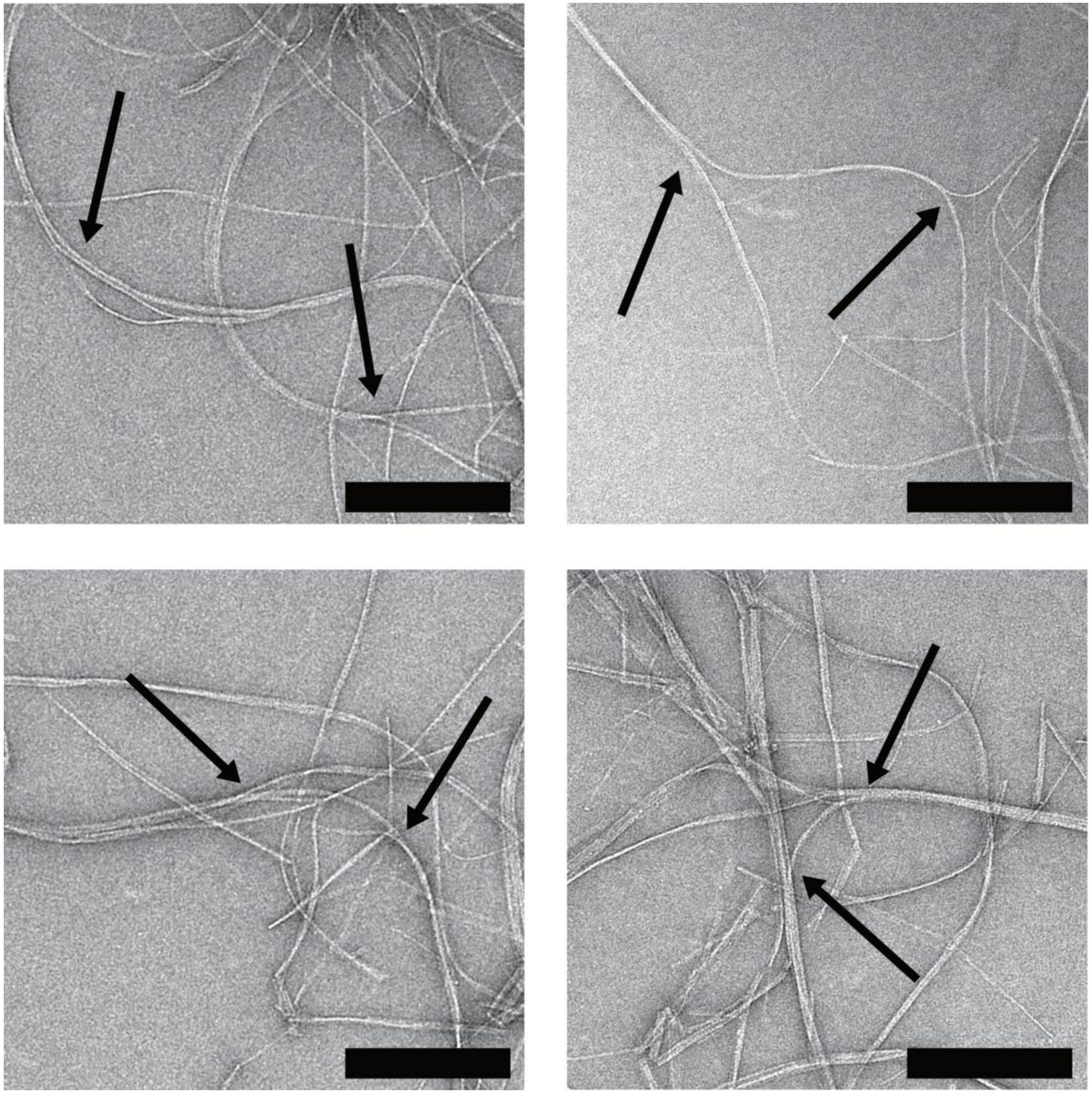

Fig. S5: The presence of 10 mM CHAPS during polymerization of wt CsgA fibrils results in significantly more disperse fibrils when imaged with TEM. This allows for a more detailed imaging of the complex lateral association of individual fibrils that results in the formation of large cable-like structures. Arrows indicate points where these cables split into thinner cables or individual fibrils. Scale bare is 250 nm.

Figure S6

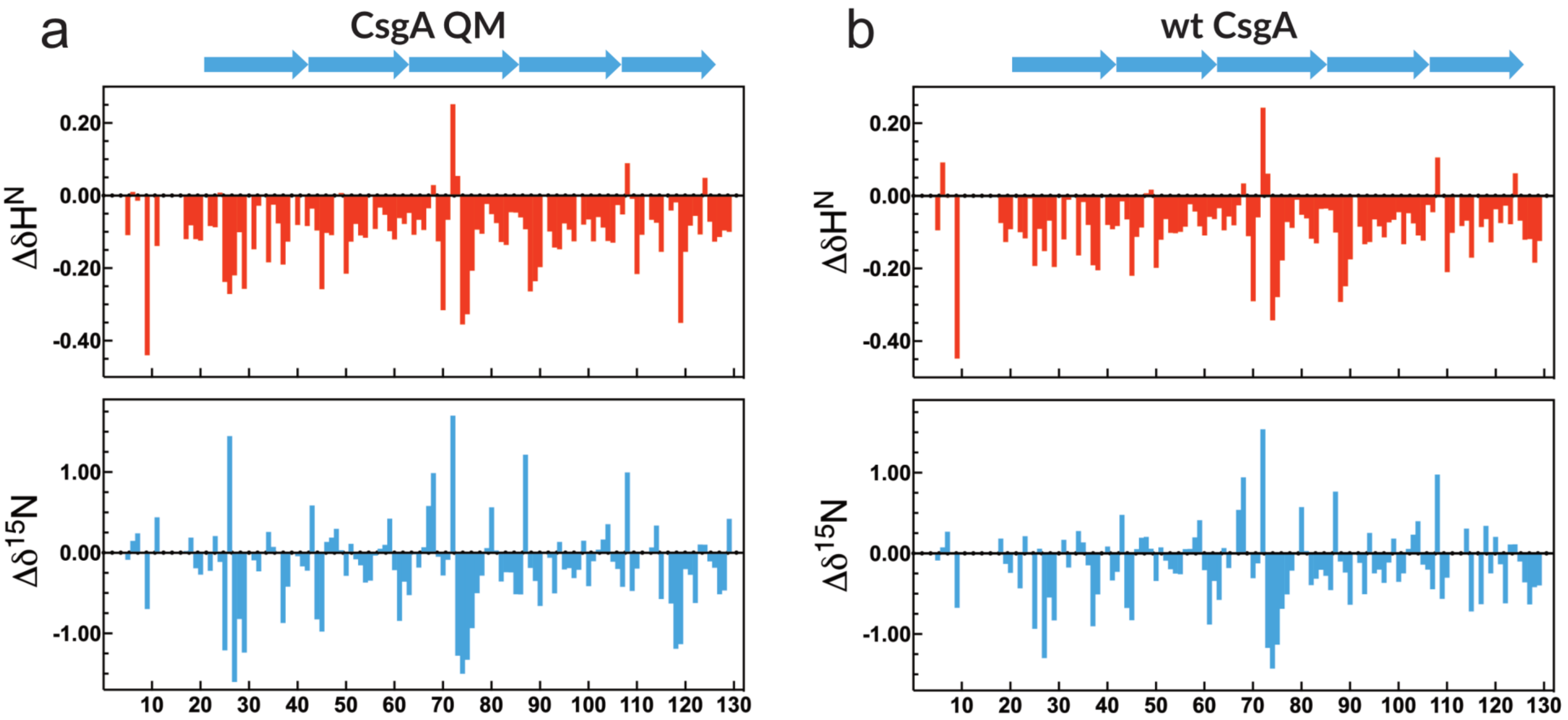

Fig. S6: ^{1}H (red) and ^{15}N (blue) secondary chemical shifts for CsgA QM (a) and wt CsgA (b) calculated by comparing assignments of the respective proteins with random coil predictions generated by POTENCI [36] ($\Delta\delta = \delta_{exp} - \delta_{POTENCI}$).

Figure S7

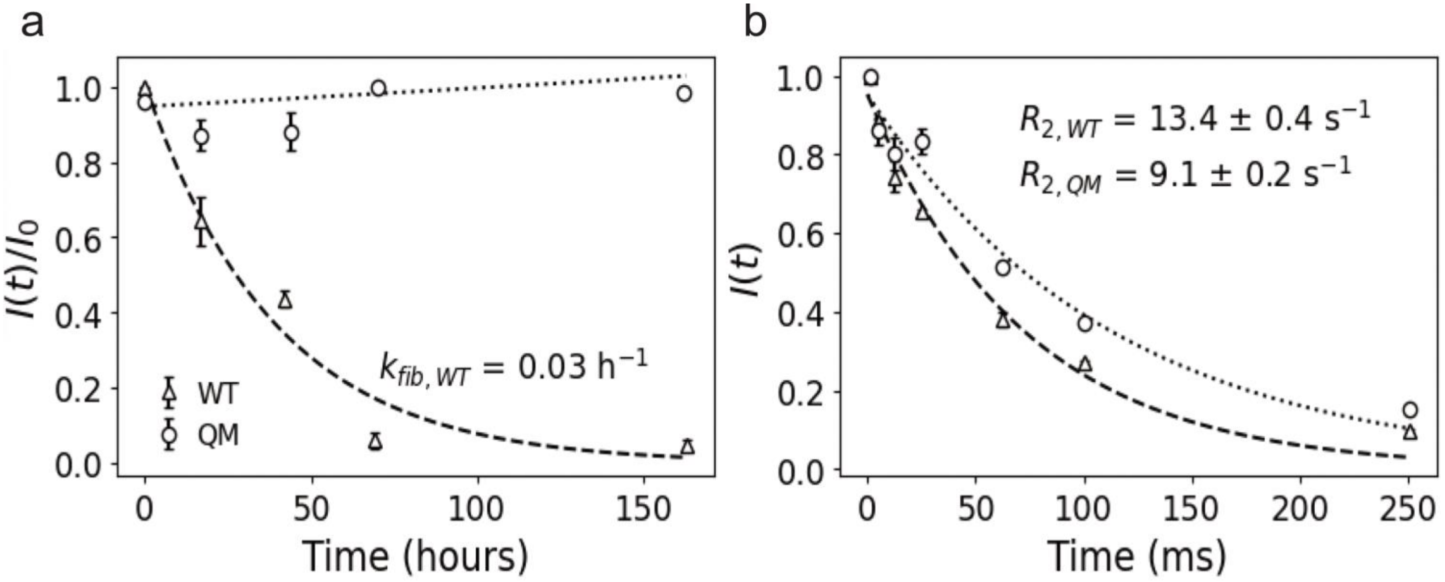

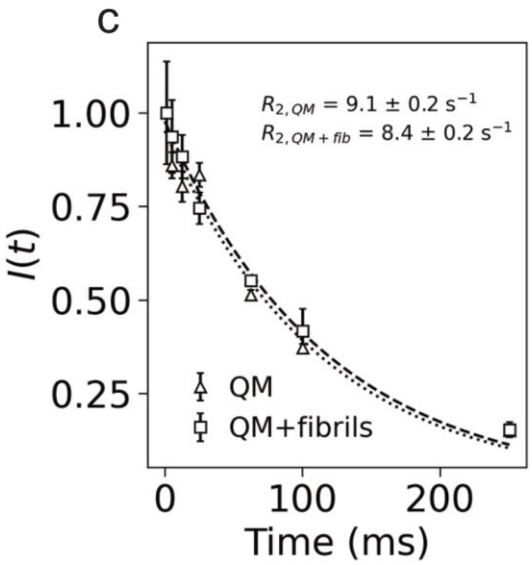

Fig. S7: (a) Signal decay of the R_2 rate for wt CsgA and CsgA QM during the 4-day experiment. (b) Estimation of transversal relaxation rates, R_2, for wt CsgA and the QM mutant. The plot shows peak integrals, I, as a function of relaxation delay, τ. The data were fit to a single exponential decay model, $I = I_0 e^{-R_2 \tau}$, to extract R_2. (c) R_2 rate for samples containing monomeric CsgA QM and monomeric CsgA QM + 5% preformed fibrils.
